# Advancements in Machine Learning-Assisted Flexible Electronics: Technologies, Applications, and Future Prospects

**DOI:** 10.3390/bios16010058

**Published:** 2026-01-13

**Authors:** Hao Su, Hongcun Wang, Dandan Sang, Santosh Kumar, Dao Xiao, Jing Sun, Qinglin Wang

**Affiliations:** 1 School of Physics Science and Information Technology, Liaocheng University, Liaocheng 252000, China; 2023404864@stu.lcu.edu.cn (H.S.); 2022406477@stu.lcu.edu.cn (H.W.);; 2 Centre of Excellence for Nanotechnology, Department of Electronics and Communication Engineering, Koneru Lakshmaiah Education Foundation, Vaddeswaram 522302, Andhra Pradesh, India; 3 School of Environmental Science and Engineering, Qilu University of Technology (Shandong Academy of Sciences), Jinan 250353, China

**Keywords:** machine learning, flexible electronics, information processing technology, intelligent flexible system

## Abstract

The integration of flexible electronics and machine learning (ML) algorithms has become a revolutionary force driving the field of intelligent sensing, giving rise to a new generation of intelligent devices and systems. This article provides a systematic review of core technologies and practical applications of ML in flexible electronics. It focuses on analyzing the theoretical frameworks of algorithms such as the Long Short-Term Memory Network (LSTM), Convolutional Neural Network (CNN), and Reinforcement Learning (RL) in the intelligent processing of sensor signals (IPSS), multimodal feature extraction (MFE), process defect and anomaly detection (PDAD), and data compression and edge computing (DCEC). This study explores the performance advantages of these technologies in optimizing signal analysis accuracy, compensating for interference in high-noise environments, optimizing manufacturing process parameters, etc., and empirically analyzes their potential applications in wearable health monitoring systems, intelligent control of soft robots, performance optimization of self-powered devices, and intelligent perception of epidermal electronic systems.

## 1. Introduction

The deep integration of flexible electronics and machine learning (ML) as cutting-edge technologies demonstrates tremendous potential and transformative capabilities to revolutionize traditional electronic devices [1]. Flexible electronic systems based on flexible materials, with their excellent flexibility, biocompatibility, and wearable nature, lay the foundation for innovative applications in areas such as health monitoring and human–computer interactions [2,3]. ML, with its powerful data analysis and pattern recognition capabilities, has achieved remarkable results in signal processing and feature extraction and has driven the intelligentization of flexible electronic systems. The fusion of these two fields has given rise to a series of breakthrough applications, providing new ideas and methods for solving perception and interaction problems in complex scenarios [4,5].

Currently, flexible electronic technology is accelerating its transition towards practical applications, ranging from fabric-based flexible fibers to intelligent upper limb exoskeletons, with applications expanding to health monitoring, intelligent control, performance optimization, etc. [2,6]. However, as application demands continue to evolve, the limitations of flexible electronic systems in practical applications have gradually emerged. Flexible sensing signals are susceptible to motion artifacts, and the complex coupling of multimodal physiological data has significantly increased the difficulty of analysis [4,7]. Traditional algorithms perform inadequately in the real-time processing of dynamic signals and cross-scenario generalization capabilities. For example, the accuracy of conventional methods in silent speech recognition is only 87.53% [5]. Additionally, the long-term stability and integration of flexible devices limit their large-scale application, and the efficient processing of massive sensing data poses higher requirements for computational efficiency [2,8]. These complex problems are receiving powerful solutions owing to the rapid development of ML technology. The success of ML in image recognition and time-series signal processing enables the automatic extraction of key features from the massive data of flexible sensing, and optimizes the accuracy of pattern recognition, which has transformative significance in improving the performance of flexible electronic systems.

For instance, ML is widely used to optimize the processing and analysis of flexible sensing signals; CNN processes tactile signals to achieve an object recognition accuracy of 99.69%; LSTM analyzes dynamic gesture classification with an accuracy of 96.2% [1]; and the residual network (ResNet) architecture processes speech features to achieve a phoneme classification accuracy of 99.5% [9]. In health monitoring, ML improves the classification balance accuracy of Huntington’s disease by 67% using elastic net regression models [7]. In human–computer interaction, transfer learning and meta-learning reduce the calibration cost for new users and improve the accuracy of sign language translation to 92.6% [8]. Moreover, ML can assist in optimizing the design of flexible material structures and device integration. For example, algorithm optimization increases the sensitivity of pressure sensors to 62 kPa^−1^, enhancing the stability and adaptability of the system [3,10].

We focus on intelligent systems formed by the deep integration of ML and flexible electronics, specifically those encompassing a complete “sensing–processing–decision” chain. This review concentrates on systems where flexible sensors and devices serve as the hardware foundation for data acquisition, and ML algorithms act as the core for intelligent information processing. By examining representative information processing technologies and their applications in four key scenarios, this review aims to establish a coherent framework for understanding the collaborative mechanisms, performance boundaries, and future evolution of ML-assisted flexible intelligent systems.

**Figure 1:**
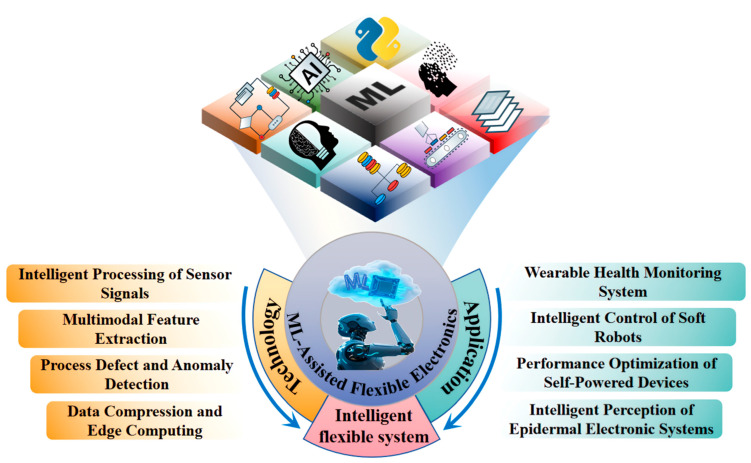
Technology and applications of machine learning-assisted flexible electronics.

This study systematically discusses the collaborative principles of ML and flexible electronics. The second part focuses on the sensing mechanism of flexible electronic materials, the basic theory of ML algorithms, and four information processing technologies combined, elaborating on their core positions and key roles in flexible intelligent systems. The third section discusses the applications of ML-assisted flexible electronics in wearable health monitoring systems, intelligent control of soft robots, performance optimization of self-powered devices, and intelligent perception of epidermal electronic systems, demonstrating how ML achieves precise analysis of flexible sensing signals and enhances the generalization ability and operational stability of the system (Figure 1). Next, this paper analyzes the current application prospects and technical challenges of ML in the field of flexible electronics, explores potential future directions and engineering implementation strategies to build a theoretical framework for subsequent research, guides practical application implementation, and promotes the field towards efficient and reliable practicalization.

Meanwhile, this review focuses specifically on the integration of ML with flexible bioelectronics, emphasizing applications where flexible sensors interface directly with biological systems for sensing and monitoring purposes. The primary scope encompasses biosignal acquisition and processing, including but not limited to: biopotentials, biochemical sensing, wearable physiological monitoring, and epidermal electronic systems for human–machine interfaces. Studies were selected based on their relevance to biosensing signal chains—from sensor design and signal acquisition to ML-enabled interpretation and decision-making. While several referenced ML techniques have broader applicability in industrial or non-biological domains, their inclusion here is justified by their demonstrated utility in enhancing biosensor performance, noise robustness, and real-time processing in flexible wearable contexts. Examples primarily drawn from non-biological domains are included only where they illustrate methodological advances directly transferable to biosensing challenges, such as anomaly detection in physiological time-series or multimodal fusion in noisy environments.

## 2. ML-Assisted Information Processing Technology for Flexible Electronics

Intelligent processing of sensor signals (IPSS), multimodal feature extraction (MFE), process defect and anomaly detection (PDAD), data compression, and edge computing (DCEC) are interrelated ML-assisted flexible electronic information processing technologies. They jointly drive the emergence of intelligent perception systems and lay the foundation for efficient and robust information acquisition and analysis in complex scenarios. Through the combination of the highly adaptable hardware of flexible sensors and the dynamic feature extraction and noise suppression capabilities of ML algorithms, the IPSS significantly improves the efficiency and accuracy of signal processing. These four methods influence and collaborate with each other. For example, high-quality signals processed by IPSS provide a reliable input data source for MFE, enabling the effective fusion and analysis of multi-source heterogeneous information. The DCEC is crucial in data reduction and real-time processing for resource-constrained scenarios, where efficient compression strategies and lightweight models complete critical computations locally, minimizing data transmission energy consumption and latency, thereby enhancing the response speed and energy efficiency of the entire system. In scenarios requiring high-precision detection, PDAD utilizes the high-resolution and high-adaptability sensing capabilities of flexible electronics to acquire multidimensional data combined with deep learning models to achieve precise identification and classification of tiny defects or anomalies. MFE leverages its cross-modal information fusion advantage to integrate multidimensional data from the IPSS, PDAD, and DCEC stages through innovative data representation and intelligent fusion model architectures, achieving more comprehensive information interpretation and decision-making in applications such as health monitoring, human–computer interaction, and intelligent control. These technologies collectively form a closed-loop processing chain, from data acquisition, processing, and compression to intelligent analysis, significantly enhancing the comprehensive performance of intelligent systems based on flexible electronics in terms of accuracy, efficiency, robustness, and adaptability.

### 2.1. Intelligent Processing of Sensor Signals (IPSS)

Traditional sensor signal processing methods have gradually revealed limitations, such as low efficiency, weak anti-interference ability, and reliance on external storage when dealing with complex dynamic scenarios. For instance, traditional tactile sensors have an accuracy rate of only 75.2% in grasping state classification [11], whereas traditional microphones have a semantic recognition accuracy rate of 33.1–46.1% in high-noise environments [12]. In this context, the integration of flexible electronics and ML provides a revolutionary solution for the intelligent processing of sensor signals, achieving significant improvements in efficiency, accuracy, and robustness through the collaboration of hardware-level feature extraction and algorithm optimization, making it a highly promising alternative. Within this review, Intelligent Processing of Sensor Signals (IPSS) is defined as a paradigm integrating flexible electronics and ML to convert noisy, dynamic raw sensor data into reliable, information-rich inputs. Central to this paradigm is a hardware-algorithm co-design philosophy. In this context, hardware-level feature extraction refers to designing materials and structures to perform intrinsic signal conditioning; multimodal data fusion describes the machine learning-driven integration of aligned signals from diverse flexible sensors to form a robust, cross-validated representation; and anti-interference design includes strategies at both the material/device level to physically suppress noise, and at the algorithmic level to computationally isolate desired signals.

**Figure 2:**
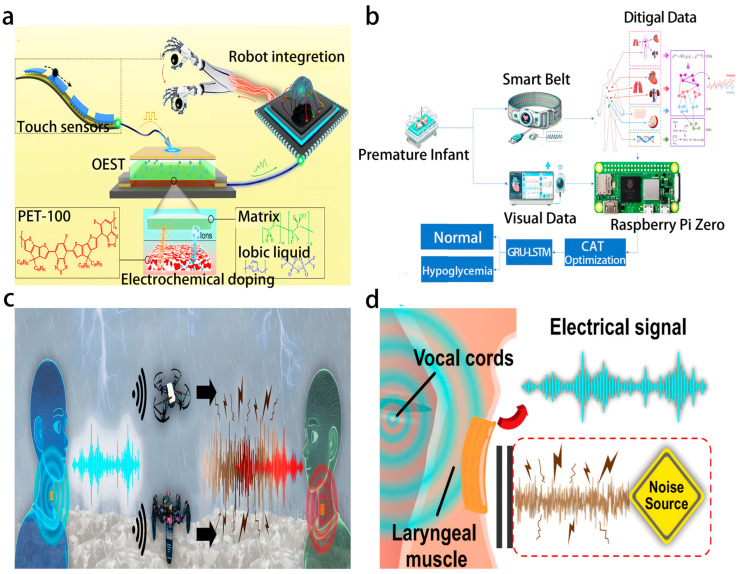
Three major breakthroughs of the ML algorithm combined with flexible electronics in the intelligent processing technology of sensor signals. (**a**) Hardware-level feature extraction. The artificial organic input neural network consists of tactile sensors and organic electrochemical transistors, and is used to collect, transmit and process tactile information in intelligent robots. Figure reproduced with permission from ref. [15]. Copyright from 2024, Springer Nature Limited. (**b**) Multi-modal data fusion with an intelligent belt and an intelligent camera equipped with a PPG sensor. Figure reproduced with permission from ref. [12]. Copyright from 2025, Springer Nature Limited. (**c**,**d**) Anti-interference designs. (**c**) The deep learning enhanced anti-noise TEAS works as a mechanism in performing complex human–machine collaboration tasks in noisy scenarios. (**d**) The anti-noise TEAS detects mixed-mode signals of acoustic signals and mechanical motion signals through contact sensing and can block the interference of environmental noise. Figure reproduced with permission from ref. [14]. Copyright from 2025, Springer Nature Limited.

The lightweight (thickness < 50 μm), flexible adhesion, and low power consumption characteristics of flexible electronics [11,13,14] provide a hardware foundation for the deployment of sensors in complex scenarios. ML algorithms achieve the precise extraction of dynamic features and noise suppression by mining the inherent patterns of signals. The integration of these two technologies has achieved three major breakthroughs: first, hardware-level feature extraction, such as the ion doping memory effect based on organic electrochemical synaptic transistors (OEST), which can complete dynamic tactile information processing without external storage (Figure 2a) [15]. Second, multimodal data fusion, which integrates multiple sources of signals such as optics, mechanics, and physiology to enhance robustness, such as the HAPI-BELT system, which integrates optical volume pulse oximetry (PPG) and visual signals to develop an intelligent belt and intelligent camera for the early detection of hypoglycemia in premature infants (Figure 2b) [12]. Third, anti-interference design, such as the nano-column structure of the anti-noise triboelectric acoustic sensor (Anti-noise TEAS), which detects mixed-mode signals of acoustic and mechanical motion signals through contact sensing (Figure 2c), can perform complex human–machine collaboration tasks in noisy environments, suppress environmental noise, and significantly improve the signal-to-noise ratio (Figure 2d) [14].

LSTM-type neural networks, with their powerful temporal modeling capabilities, excel in dynamic signal processing by addressing the long-term dependencies in sensor signals and capturing the dynamic features in the time dimension. The AOAN system developed in 2024 [15] combines OEST and LSTM to achieve closed-loop processing of robot tactile signals, with an accuracy rate of 98.7% (rising to 99.0% after 50 training sessions) for the identification of sliding directions and classification of grasping states. In the safety field, the improved Long Short-Term Memory (ILSTM) framework combined with the Crocodile Optimization Algorithm (COA) [16] achieved an accuracy rate of 98.9% for identifying vehicle network attacks, significantly enhancing defense capabilities against zero-day attacks. Additionally, the Attention-LSTM model [17] integrates UWB and INS sensor data to achieve a positioning accuracy of 0.08–0.17 m in non-line-of-sight environments, providing robust support for autonomous driving systems.

CNNs demonstrate efficient feature extraction capabilities in image, acoustic, and multimodal signal processing and are capable of uncovering local spatial correlations in signals and achieving hardware-level feature compression. The 1D-CNN-based smart hybrid fabric wristband [13] processes six-channel capacitance signals and achieves an accuracy rate of 96.63% for virtual handwriting recognition of 26 letters. The anti-noise TEAS system [14] uses a CNN to analyze laryngeal vibration signals and achieves a semantic recognition accuracy of over 99% in high-noise environments, thereby providing reliable interaction for post-disaster rescue robots. In the field of machine vision, a programmable photoelectric sensor [18] based on van der Waals heterogeneous structures integrates CNN and directly processes images with a compression ratio of 40:1, achieving a human action classification accuracy of 93.18% and significantly reducing the data transmission load.

Owing to their low-latency characteristics, gated recurrent unit (GRU)-type models dominate real-time physiological signal processing. The PHTNet model [19] uses a bidirectional GRU to separate pathological hand tremors from voluntary movements, achieving real-time compensation without phase lag and driving the development of robot rehabilitation equipment. The HAPI-BELT system [12] innovatively employs a GRU-LSTM hybrid network, integrating PPG and visual signal detection for the early detection of low blood sugar in premature infants, with an accuracy rate of 99.6%, providing a non-invasive solution for neonatal monitoring.

Improved Kalman filtering algorithms have achieved remarkable results in reducing computational complexity. The MKFs framework [20] reduces the sampling rate and dynamic optimization dictionary to decrease the computational load of high-speed communication carrier tracking by more than 80%. The KalCo framework [11] enables unsupervised feature extraction and provides a lightweight solution for high-throughput scenarios. The underwater sensor network [21] combines the improved chemical reaction optimization (CRO) algorithm to achieve a coverage rate of 95.66% and a data transmission success rate of 95.35%, thereby optimizing the efficiency of marine monitoring.

Specific optimization algorithms enhance the overall performance by integrating them with neural networks. Collaboration between COA and ILSTM [16] significantly enhances network security detection. The intelligent glasses integration system [22] utilizes multimodal large models and the Natural Language Processing (NLP) technology to achieve physiological signal analysis and personalized health advice generation, thereby promoting the development of active health management. Moreover, the augmented reality soft electrode array [23] combines support vector machines (SVM) to process electromyogram signals, achieving a classification accuracy of 96.08% for 10 gestures.

The integration of flexible electronics and ML overcomes the bottlenecks of traditional sensor signal processing, demonstrating outstanding performance in terms of accuracy, efficiency, and robustness. In the future, with the development of edge computing and multi-modal fusion technology, this field will provide more efficient and reliable signal-processing paradigms for scenarios such as intelligent robots, digital health, and autonomous driving.

### 2.2. Multimodal Feature Extraction (MFE)

The integration of ML with flexible electronics provides an innovative solution for multimodal feature-extraction technology. Owing to the high adaptability of flexible sensing and the intelligent processing capabilities of ML, it is one of the core technologies in the field of multimodal information processing, playing an important role in health monitoring, human–computer interaction, and medical diagnosis. This technology captures multidimensional physical, chemical, or physiological signals through flexible electronic devices and realizes feature fusion and analysis through ML algorithms, significantly improving the accuracy and efficiency of information interpretation in complex scenarios. It can handle various modal data and optimize the entire process, from signal capture to intelligent decision-making, through multi-sensor collaborative collection and cross-modal modeling.

In terms of hardware-level multimodal integration and sensor innovation, the adaptability of flexible electronics and the pattern recognition ability of ML form an efficient synergy. In recent years, Qiu et al. developed a liquid metal (LM) flexible sensing system [24] that uses SiO_2_ microparticles to regulate the rheological properties of gallium-indium alloys for 3D printing to fabricate sensors with both strain and pressure sensing capabilities. Combined with convolutional neural network (CNN) processing of multimodal signals from boxing actions, the recognition accuracy reached 90.5%. Wen et al. developed a seamless integrated structure wearable multimodal sensor [25] that decouples strain and pressure signals based on resistance-capacitance components and processes them through LSTM neural networks, achieving a recognition accuracy of 97.13% for 10 joint postures, significantly outperforming a single modality. Choi et al. developed a fully printed chipless wearable neuromorphic system [26], integrating metabolites, body temperature, and pressure sensors, and achieved a classification accuracy of 84.4% through neurocomputational methods for sepsis classification, demonstrating the high-performance advantage of hardware-algorithm synergy. Additionally, bio-inspired composite hydrogels [27] serve as flexible electrodes for high-precision muscle signal acquisition, supporting stable monitoring for 48 h and providing hardware support for multimodal biological feature extraction.

In terms of cross-modal data representation and conversion methods, ML achieves heterogeneous modality fusion through innovative data representations. Tian et al. proposed a dynamic bone recognition method for spinal plate cutting robots [28] using six-dimensional force sensors to collect cutting force signals and combining PSO-SVM algorithms, achieving a bone layer recognition accuracy of 90.64% and an improvement of 7.08% over traditional SVM. Moreover, the deep learning method based on multi-modal physiological signal image transformation [29] converts Electrodermal Activity (EDA), Accelerometer (ACC), and other signals into RGB images and uses CNN processing to achieve stress state classification, with an accuracy of 90.96%. The multimodal malware classification method [17] integrated numerical and image data, achieving an accuracy of 95.36%. The few-shot network intrusion detection system [30] combined traffic feature maps and network feature sets, achieving an accuracy of 98.50%.

Within the domain of multimodal sensing systems described here, the terms “3D” and “six-dimensional” refer to distinct technical attributes. “3D” primarily pertains to fabrication and structural geometry, specifically denoting three-dimensional printing techniques that enable the construction of flexible sensors with complex spatial architectures Conversely, “six-dimensional” describes the measurement capability of a sensor, typically referring to a force/torque sensor that can simultaneously detect forces along three orthogonal axes (x, y, z) and torques around these three axes, thereby providing a complete description of mechanical interaction forces. This distinction underscores the complementary nature of advanced manufacturing (3D) for creating sophisticated sensor forms and advanced sensing (six-dimensional) for capturing richer physical interaction data, both crucial for enhancing multimodal feature extraction.

**Figure 3:**
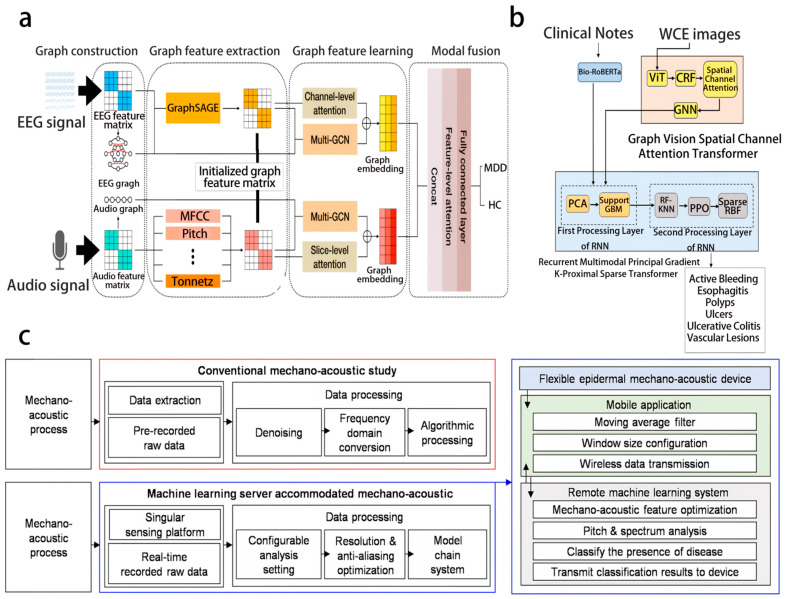
Three advanced neural network architectures for multimodal feature extraction technology. (**a**) The framework of EMO-GCN. Figure reproduced with permission from ref. [31]. Copyright from 2024, Springer Nature Limited. (**b**) The framework for gastrointestinal disease classification. Figure reproduced with permission from ref. [32]. Copyright from 2025, Springer Nature Limited. (**c**) Comparison with traditional mechanical acoustic analysis methods; this shows the framework of mechanical acoustic signals entering the machine learning algorithm. Figure reproduced with permission from ref. [33]. Copyright from 2024, Springer Nature Limited.

Advanced neural network architectures enable end-to-end feature learning and decision-making in the design of intelligent fusion model architectures. Xing et al. proposed the EMO-GCN adaptive multi-graph neural network framework (Figure 3a) [31], combining EEG and audio signals and using channel-level and slice-level attention mechanisms to fuse features, achieving an accuracy of 96.76% in MDD detection, which is superior to single-modal methods. Additionally, the gastric disease classification framework proposed by Sharmila et al. (Figure 3b) [32] integrates wireless capsule endoscopy images and medical text using a cross-attention mechanism to align features, achieving a classification accuracy of 99.82% and providing a new tool for non-invasive diagnosis. Moreover, the real-time deep learning-assisted mechanical voice system (Figure 3c) [33] captured throat mechanical voice signals through flexible devices, and the multimodal model achieved an accuracy of 93% for COPD classification and 95% for word recognition.

The development of this technology represents a significant breakthrough in the integration of flexible sensing and intelligent algorithms, thereby opening up new paths for cross-domain applications. Compared with traditional single-modal systems, it has significant advantages in terms of adaptability to complex environments [34], robustness of signal analysis [35], and utilization of multidimensional information [34,36,37]. These advancements have promoted the continuous optimization of multi-modal feature extraction technology in the intersection of flexible electronics and ML, providing efficient solutions for scenarios such as personalized medicine, intelligent industry, and human–computer interaction.

### 2.3. Process Defect and Anomaly Detection (PDAD)

Process defect anomaly detection is a crucial step in ensuring product quality and service safety in modern manufacturing, healthcare, and other fields. Traditional detection methods rely on manual screening or single-sensing technologies, which have problems such as low efficiency, high missed detection rate, and poor adaptability. In particular, when dealing with tiny defects and multidimensional data in complex environments, they perform poorly. For example, tiny defects (0.1 mm on the surface of chips), various types of defects in PCB manufacturing, or subtle abnormal fluctuations in vital signs during medical monitoring are difficult to accurately capture [38,39,40]. To address these challenges, the combination of ML and flexible electronics has become an innovative technological solution that overcomes bottlenecks. By providing a highly adaptable sensing platform through flexible electronics and combining the powerful data processing capabilities of ML, it achieves efficient perception and precise identification of defect information in complex scenarios, thereby providing a new information processing paradigm for process defect anomaly detection.

The core of the integration of ML and flexible electronics lies in the “sensing–processing” collaborative mechanism. Flexible electronic technology provides a highly adaptable hardware foundation. Wearable sensors, industrial camera arrays, etc., provide multi-dimensional information such as high-resolution images, physiological signals [40], and multi-spectral data [41]. This overcomes the adaptation problems of traditional sensors in complex and dynamic environments [42]. The ML algorithm serves as the core of information processing, feature extraction, and pattern recognition through methods such as deep learning and traditional statistical learning. For example, CNN can capture spatial details in images [38,43], the transformer module enhances global feature dependency modeling, and LSTM autoencoders achieve abnormal reconstruction of time-series signals [40]. The combination of these significantly improves the accuracy and processing efficiency of the defect information.

**Figure 4:**
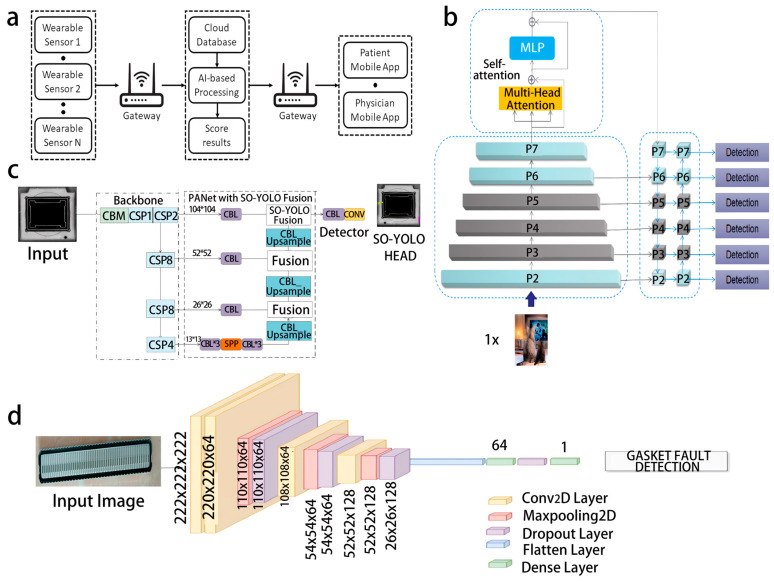
Framework and mechanism for process defect anomaly detection. (**a**) Overall architecture of the proposed soft sensor. Figure reproduced with permission from ref. [40]. Copyright from 2022, Springer Nature Limited. (**b**) Design of ATT-YOLO. Figure reproduced with permission from ref. [41]. Copyright from 2023, Springer Nature Limited. (**c**) Architecture of the proposed SO-YOLO. Figure reproduced with permission from ref. [38]. Copyright from 2022, Springer Nature Limited. (**d**) Hierarchical view of the deep convolutional neural network architecture. Figure reproduced with permission from ref. [43]. Copyright from 2025, Springer Nature Limited.

In practical applications, this collaborative advantage is reflected in three aspects. First, the high adaptability of flexible electronics expands the data collection range. For example, wearable sensors have been adapted for human motion monitoring (Figure 4a) [40], and Attention mechanism You Only Look Once (ATT-YOLO) uses multi-scale backbone networks and feature pyramids to cover the entire surface of electronic components with multi-spectral industrial cameras (Figure 4b) [41]. Second, the intelligent processing ability of ML enables the precise classification of defects. For example, Solder Object You Only Look Once (SO-YOLO) distinguishes “edge loss” and “excess solder” in chips (Figure 4c) [38], and Transformer and convolutional networks identify “short circuit” and “spurs” in Printed Circuit Boards (PCBs) [39]. Third, a combination of the two supports real-time detection. Lightweight models of deep convolutional neural networks (Figure 4d) [43] combined with efficient sensing transmission meet the real-time requirements of production lines or medical monitoring.

Collaborative optimization of flexible imaging and deep learning architectures has overcome the defect detection challenges in the electronics manufacturing field. Huang et al. proposed a chip surface defect detection scheme based on a shallow feature fusion network [38] by using a flexible industrial camera to capture high-resolution images, combining mosaic data augmentation to expand the dataset, and utilizing the improved PANet structure to fuse shallow features while retaining the spatial details of minor defects. By combining k-means++ optimized anchor boxes, the model achieved an mAP of 86%, significantly outperforming the traditional YOLOv4 (82.6%), and successfully identifying defects as small as 0.1 mm. For PCB manufacturing defects, an end-to-end deep learning framework was designed using an automatic optical inspection (AOI) system to collect images, reduce hardware requirements through double cubic downsampling, and integrate the advantages of CNN and Transformer, with an mAP of 98.1% and only 7.02 M parameters, achieving a lightweight design suitable for high-speed production lines. The ATT-YOLO model introduced a self-attention module [41], combined with notebook computer shell images collected by multispectral flexible sensing, through image slicing technology processing 5000 × 5000 resolution data, achieving a multiclass classification mAP of 90.3%, with a 111 FPS inference speed, meeting the real-time detection requirements for surface defects of electronic components.

The closed-loop design of flexible biosensors and time-series anomaly detection algorithms is innovative. The soft sensor remote monitoring system developed by the Arpaia team integrates the MAX30100 flexible sensor (Maxim Integrated, San Jose, CA, USA) and ESP32 microcontroller (Espressif Systems, Shanghai, China) [40], transmitting heart rate and blood oxygen data to the cloud via the MQTT protocol, combining multiple linear regression (MLR) to estimate blood pressure, using an LSTM autoencoder to build an anomaly detection model, training with 80% normal data to personalize thresholds, achieving an anomaly detection accuracy of 93% and an F1 score of 0.96, and providing a personalized solution for remote monitoring of patients with chronic diseases. Additionally, the SDN-IoT medical intrusion detection framework proposed by the Arthi team [44] integrates wearable flexible sensors and DNN-SVM hybrid models, extracting 44-dimensional network traffic features from DNN and implementing attack behavior classification with SVM, achieving an F1 score of 0.96, ensuring the security of medical IoT data transmission.

The improvement of flexible perception and model robustness in extreme environments. Suh et al. targeted 3D geometric steel surface defects [42], using a multi-camera flexible vision system to collect images, compensating for imaging distortion through perspective transformation, designing a lightweight CNN model (5 convolutional layers), with an average F1 score of 0.932, suitable for low-quality image detection in 800–1200 °C high-temperature environments. Additionally, EBSM additive manufacturing component defect detection [45] combined X-ray CT scanning and selective box fusion (SBF) integration methods, achieving a defect detection mAP50 of 0.7594 for the detection of cracks, incomplete fusion, etc., improving by 3% compared to a single model. The CNN model for gasket fault detection by Arumai et al. [43] collected images using a high-resolution industrial camera, trained after data augmentation (rotation, noise addition, etc.), with an accuracy rate of 97.32%, and capable of quickly identifying assembly defects such as misalignment and improper installation.

Although this technology system has achieved significant progress, practical applications still face many challenges: difficulties in extracting features of minor defects, data quality degradation owing to complex environments, and the contradiction between model calculation cost and real-time requirements. Researchers have gradually overcome these limitations through targeted optimization. At the data level, enhanced technologies, such as mosaic fusion, geometric transformation, and HSV adjustment, are adopted to expand the dataset and enhance the generalization ability of the model, and shallow feature fusion and self-attention modules are introduced to enhance the capture of minor defects and global features. At the model design level, lightweight models are achieved by removing redundant branches and simplifying the number of network layers, such as the model in [39] with only 7.02 M parameters, which is suitable for high-speed production lines. At the algorithm fusion level, hybrid models (DNN + SVM) and ensemble methods were used to balance the detection accuracy and efficiency.

However, some methods still have limitations. For example, the SBF ensemble method [45] has limited improvement in the detection of fine pores; the CNN model requires further verification of its robustness under extreme lighting conditions. In the future, more refined feature engineering and adaptive algorithms should be combined to enhance adaptability to complex scenarios. Overall, the combination of ML and flexible electronics provides an efficient information processing technology solution for process defect anomaly detection, improving the detection accuracy and efficiency through the “sensing–processing” collaboration.

### 2.4. Data Compression and Edge Computing (DCEC)

In the field of information processing, a new solution has been provided for data compression and edge computing in resource-constrained scenarios by integrating the efficient data acquisition capability of flexible electronics with the intelligent processing capability of ML. This hybrid method utilizes the lightweight and conformal characteristics of flexible electronics to achieve precise data capture in complex scenarios while leveraging ML algorithms to optimize data compression strategies and edge inference efficiency, breaking through traditional hardware constraints and computational resource limitations. Its basic framework includes collecting raw data through flexible sensors, reducing redundant information through data compression techniques, and completing feature extraction and inference in edge devices using ML models, ultimately achieving low-latency and low-power real-time information processing. Different from the traditional architecture that relies on rigid equipment and cloud computing, this paradigm adopts a closed-loop design of “collection–compression–local processing,” taking into account both data integrity and processing efficiency, and demonstrating unique advantages in medical diagnosis, motion monitoring, and industrial sensing fields. The “collection–compression–local processing” closed-loop framework is an intentional architectural design that systematically addresses the core constraints of resource-limited scenarios; it is this very structure that gives rise to the key advantages—low latency, minimal power consumption, and robust real-time performance—detailed throughout this section.

The developmental history of this technology system shows the characteristics of collaborative innovation in multiple fields. In 2021, Dong et al. combined ML and data compression technology in the J-TEXT tokamak device using down-sampling technology (reducing the sampling frequency to 100 kHz) for plasma diagnostic signals to reduce data volume and through the GRNN model to achieve electron temperature prediction. The single-channel calculation time is only 7.63 s [46], significantly adapting to the real-time requirements of edge computing and laying the foundation for subsequent cross-domain applications of data compression and lightweight models.

**Figure 5:**
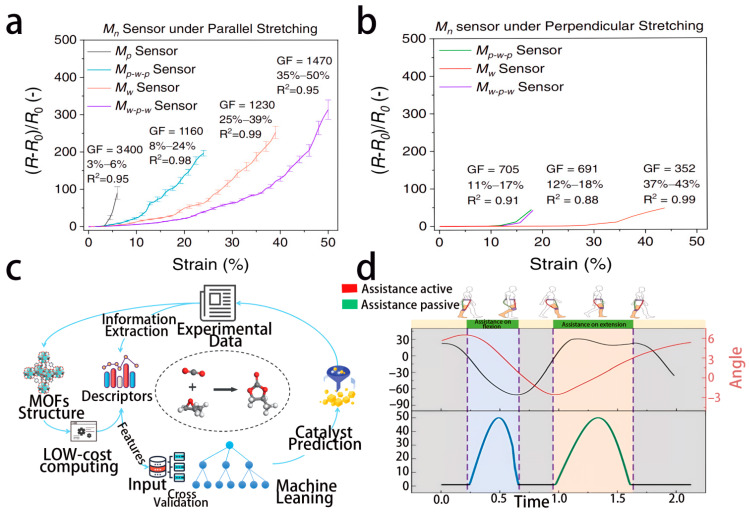
The framework and mechanisms formed by data compression and edge computing in recent developments. (**a**,**b**) In 2022, the Yang research team made a breakthrough in the field of flexible electronics with edge computing technology. (**a**) The relative resistance change strain curve of the Mn sensor under parallel stretching. (**b**) The gauge factor and linear working window relative resistance change strain curve of the Mn sensor under vertical stretching, with the edge module integrated into the ML chip. Figure reproduced with permission from ref. [48]. Copyright from 2022, Springer Nature Limited. (**c**) In 2024, the Bai team developed a workflow for machine learning model training and high-throughput screening of MOF catalysts. Figure reproduced with permission from ref. [50]. Copyright from 2025, Elsevier. (**d**) In 2025, the Sun team developed the bending and stretching mechanism of the flexible exoskeleton. Figure reproduced with permission from ref. [52]. Copyright from 2025, Springer Nature Limited.

In 2022, two key studies promoted the integration of flexible electronics and ML. For the classification of tremors in multiple sclerosis (MS), Hossen et al. used 2 g lightweight accelerometers and surface electromyography electrodes to collect physiological signals, processed 800 Hz sampled data through Soft Decision Wavelet Decomposition (SDWD) technology, extracted power spectral density features, and combined support vector regression (SVR) to achieve tremor-type differentiation. The accuracy rates of accelerometer and EMG data classification reached 91.67% and 91.60%, respectively [47], confirming the feasibility of combining flexible sensing with fine signal processing. In the same year, a wearable flexible sensor module based on MXene was developed, achieving a 6–84% adjustable working window through terrain design, suitable for multi-joint motion monitoring. Its edge module integrated an ML chip, using CNN for local data processing, achieving full-body reconstruction, reducing power consumption by 71% compared to wireless transmission mode (Figure 5a,b) [48], indicating a breakthrough in the practical application of edge computing to flexible electronics.

In 2024, technology application was extended to more complex scenarios. In the edge assessment of basal cell carcinoma surgery, Levy et al. targeted the high-resolution characteristics of full-section images, using 256 × 256 pixel block technology to screen effective tissue regions and processing multi-channel data through a parallel computing framework, combining a CNN-GNN hybrid model to achieve tumor localization with a single-case processing time of only 78 s, reducing 80% of the time consumption compared to serial processing [49], verifying the efficiency of data blocking and parallel computing in edge environments. At the same time, in the high-throughput screening study of MOF catalysts, a low-computational-cost descriptor combined with a random forest model was used to screen 12,515 structures per day on an ordinary computer, with a test accuracy rate of 98.65% (Figure 5c) [50], thereby providing a reference for feature engineering in low-resource scenarios.

By 2025, this field had witnessed multiple technological innovations. The hybrid deep learning image compression architecture achieves region-adaptive compression through a static wavelet transform and gray-level co-occurrence matrix and combines a stacked denoising autoencoder to achieve 0.065-s encoding and decoding on an NVIDIA T4 GPU (70 W). The peak signal-to-noise ratio reaches 50.36 dB, which is suitable for edge storage and the transmission of medical images [51]. In the field of flexible exoskeletons, hip joint flexion and extension assistance systems use elastic materials to construct actuators. It uses the STM32F429 chip and ESP32 unit as edge devices to process tension and angle signals in real time, combines the TD3 algorithm to optimize PID control, and reduces the metabolic cost of walking on flat ground by 12.9% ± 3.3% (Figure 5d) [52], demonstrating the potential of edge intelligence for dynamic control. Moreover, in precise oncology research, the combination of biological sensors and federated learning enables multi-institutional collaboration, with the model trained locally and only sharing parameters, balancing privacy protection and data utilization efficiency [53], further expanding the ethical adaptability of the technology.

Notably, machine learning also demonstrates significant potential in optimizing the intrinsic properties of flexible electronic materials, particularly in material systems lacking well-defined physical mechanisms. For instance, Zhang et al. developed a dielectric constant prediction and reflection optimization system, which employed a Random Forest regression model to predict the electromagnetic properties of flexible BaTiO_3_ composites based on key processing parameters [54]. By establishing a predictive model with limited experimental data and subsequently expanding the database, the study facilitated the design of a dual-layer impedance gradient structure, ultimately achieving ultra-broadband absorption from 3.29 to 18 GHz with a minimum reflection loss of −56.08 dB at a thickness of 10 mm. This work illustrates that ML is not only applicable to sensor signal processing and system control but can also construct an inverse mapping between “process parameters–material properties,” significantly accelerating the development of high-performance flexible functional materials. It provides an exemplary case of intelligent design at the material level for realizing complex multifunctional flexible systems.

**Figure 6:**
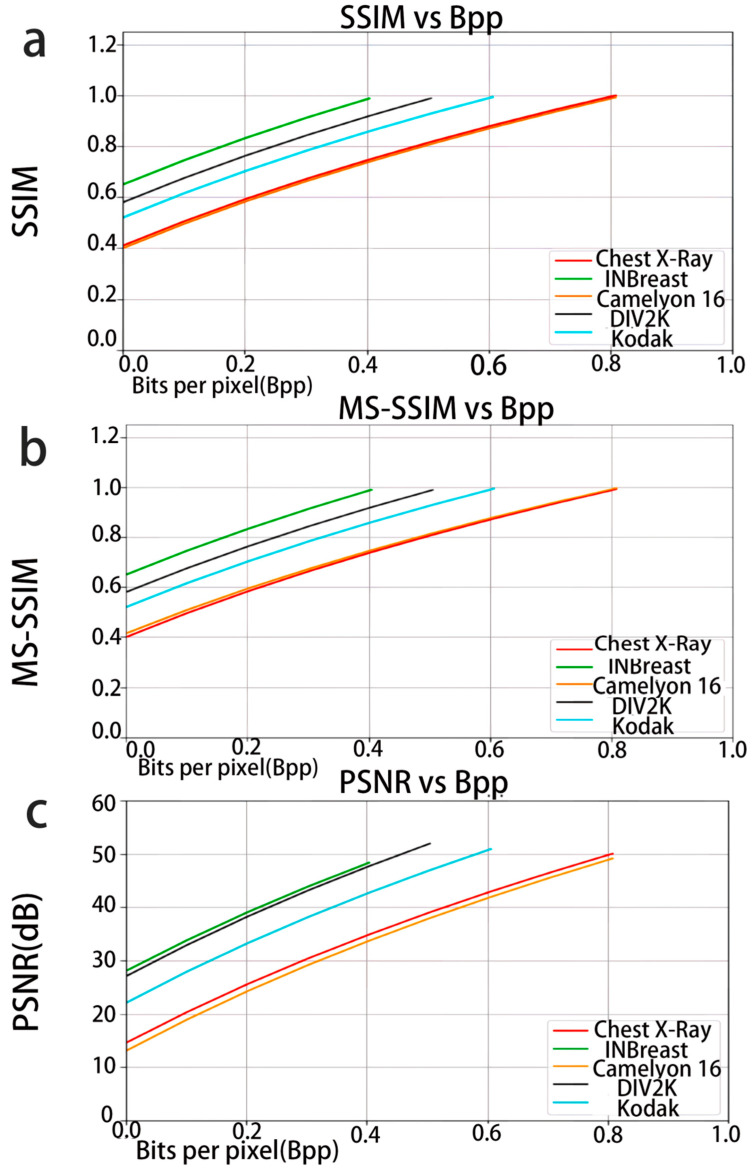
Six standard evaluation indicators were adopted, and the quantitative evaluation of the proposed model on five datasets was presented, achieving a high compression ratio for the data. (**a**) The comparison of the structure similarity between peak signal-to-noise ratio and per-pixel bit number at different compression levels, with the score approaching 1.0. (**b**) The comparison of the multi-scale structural similarity index with per-pixel bit number. This model consistently achieves a peak signal-to-noise ratio of over 48 decibels, outperforming previous methods. (**c**) The comparison of the structural similarity index with the per-pixel bit number. This confirms its superior perceptual quality. Figure reproduced with permission from ref. [51]. Copyright from 2025, Springer Nature Limited.

The latest developments indicate that this technical system has achieved significant breakthroughs in terms of data compression strategies, edge hardware collaboration, and model robustness. Data compression technology has evolved from a single downsampling approach to a multi-modal strategy, including differentiated downsampling based on signal characteristics [46] and block filtering based on image content. In addition, as shown in Figure 6, Adaptive clustering based on texture features assesses multiple datasets to ensure the robustness and universality of the model. Comparative results with traditional and state-of-the-art compression techniques demonstrate that this model can achieve high compression ratios while maintaining key image quality [51]. Edge computing optimization focuses on hardware–algorithm collaboration. Through dedicated chip integration, low-power architecture design, parallel computing scheduling, and local inference latency, power consumption is reduced by more than 50% compared to traditional solutions. In terms of model adaptation, lightweight design, reinforcement learning optimization, and interpretation ability enhancement have improved generalization ability and reliability in complex environments.

In summary, the four information processing technologies demonstrate significant performance improvements across various application scenarios. To systematically summarize and compare the performance of different models in key tasks, the main evaluation metrics are summarized in Table 1, providing a clear overview of the current performance and technological trends of machine learning in flexible electronics. And to elucidate their distinct yet complementary roles in advancing biomedical applications, Table 2 provides a synthesized comparison, highlighting biosensing-specific advancements, inherent advantages, persisting challenges, and representative use cases. This analysis underscores how the co-design of flexible sensing hardware and intelligent algorithms is tailored to address core demands in health monitoring, diagnosis, and personalized medicine.

## 3. Applications of ML in Flexible Electronics

With its strong capabilities in data parsing, pattern recognition, and adaptive optimization, ML has deeply integrated into the development process of flexible electronic technology, becoming the core driving force that promotes breakthroughs in this technology in areas such as health monitoring, robot control, energy management, and epidermal electronics. As mentioned in Section 2, flexible electronics, with their flexibility, biocompatibility, and wearability, have overcome the application limitations of traditional rigid electronics. ML, through intelligent processing of multidimensional and high-noise data collected by flexible sensors, accurately converts raw signals into valuable information. The deep integration of these two elements is key to the practicalization and intelligence of flexible electronic technology. This section will explore the core role of ML in four typical flexible electronic application scenarios: wearable health monitoring systems, intelligent control of soft robots, performance optimization of self-powered devices, and intelligent perception of epidermal electronic systems. These scenarios provide a solid foundation for the widespread promotion of flexible electronic technology. 

### 3.1. Wearable Health Monitoring System

The integration of flexible electronic technology and ML provides a revolutionary impetus for the development of wearable health-monitoring systems. Flexible electronics, with their high flexibility, biocompatibility, and portability, overcome the limitations of traditional rigid electronic devices in terms of human adaptability, while ML achieves precise mapping from physiological signals to health status through intelligent analysis of multi-dimensional sensing data. In recent years, research in this field has covered multiple scenarios such as sports rehabilitation, chronic disease management, and infectious disease monitoring, forming a complete technical chain of “flexible sensing–data transmission–intelligent analysis–health intervention” [55,56,57].

**Figure 7:**
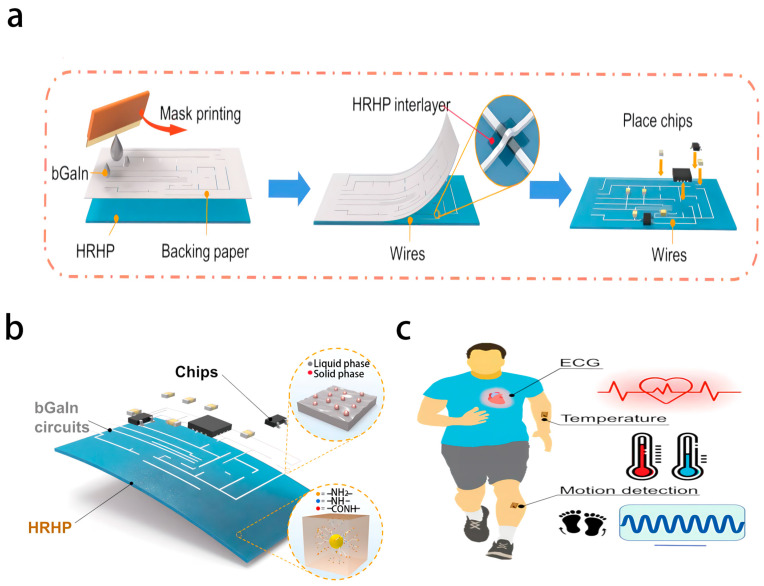
Self-repairing flexible electronic devices for health monitoring. (**a**) Schematic diagram of the manufacturing process of the self-healing electronic device. (**b**) For health monitoring. (**c**) Decomposition diagram of the concept-verified multi-functional electronic device for electrophysiology, temperature and motion. Figure reproduced with permission from ref. [58]. Copyright from 2023, John Wiley & Sons, Inc.

Flexible electronic materials and microfabrication technology are the core supports of wearable health-monitoring systems. Existing research uses diverse flexible substrates and combines conductive materials to construct sensors, enabling noninvasive and continuous collection of physiological signals. In terms of material selection, textile-based sensors achieve synchronous monitoring of the electrocardiogram (ECG) and sweating rate by embedding graphene-functionalized fabric electrodes into the fabric, with superior breathability and wear comfort compared to traditional electrodes. Electronic tattoos adopt a graphene/PMMA double-layer structure, with an elongation exceeding 40% and transparency reaching 85%, and can directly adhere to the skin to detect bioelectric potentials, such as electromyography (EMG) and electroencephalography (EEG) [55]. The sensing mechanism is based on the establishment of a stable electrochemical interface between the conductive layer and the electrolyte-rich stratum corneum of the skin. Ionic currents generated by underlying muscle or neural activity are transduced into measurable electronic signals at this interface. The high conformability and stretchability of the structure maintain consistent contact impedance during skin deformation, thereby minimizing motion artifacts and ensuring high-fidelity signal acquisition. Liquid metals are fabricated through mask printing to achieve a tensile strength of over 900%, a conductivity of 3.40 × 10^4^ S/cm, and adaptability to signal stability in intense exercise scenarios [8,58]. In terms of fabrication techniques, screen printing and inkjet printing have enabled the integration of multiparameter sensing arrays. For example, the flexible integrated sensing array (FISA) monitors electrolytes such as glucose, lactic acid, Na^+^, K^+^, and Cl^−^ in sweat through ion-selective electrodes, combined with microfluidic structures to achieve non-invasive sampling, with a lightweight sampling process and minimal burden on users [59]. The operational principle of FISA involves capillary-driven microfluidic transport of sweat to dedicated sensing chambers. Each chamber houses an ion-selective electrode with a membrane containing specific ionophores. For ion detection, the potential difference across the membrane, governed by the Nernst equation, correlates with the ion concentration. For metabolite detection, an enzymatic reaction (e.g., catalyzed by glucose oxidase) produces electroactive by-products, which are subsequently quantified via amperometry. This integrated design enables real-time, multiplexed analysis of sweat biomarkers. Additionally, the introduction of self-healing materials further enhances the durability of the equipment, such as a substrate based on hyperbranched polymers (HRHP) that can recover 5.5 a of tensile strength within 1 min through hydrogen bond exchange and restore circuit functionality within 5 min after fracture (Figure 7) [58].

A compelling demonstration of such an integrated system is the development of a microfluidic wearable electrochemical sensor for multiplexed sweat analysis. Recent work has seamlessly combined a 3D-printed microfluidic chip with flexible electrochemical sensors fabricated using MOF-derived hexagonal rod-shaped porous carbon [60]. This design addresses key challenges in wearable monitoring: the microfluidic chip enables rapid, contamination-free sweat sampling, while the PCN-based working electrode provides high sensitivity and selectivity for metabolite detection. Simultaneously, integrated ion-selective electrodes allow for the potentiometric detection of electrolytes like K^+^ and pH. Crucially, the system maintains stable electrochemical performance under mechanical deformation, confirming its robustness for on-body applications. This platform successfully facilitated the real-time, correlative analysis of sweat uric acid, pH, and K^+^ during exercise, and further investigated the relationship between sweat and urine metabolite levels. This example underscores how the convergence of advanced nanomaterials, microfluidics, and flexible electronics can translate into practical, multifunctional devices for non-invasive, dynamic health monitoring, paving the way for personalized physiological insights.

ML, through feature extraction, pattern recognition, and predictive modeling, has solved the problems of noise interference, individual differences, and multiparameter coupling in flexible sensor data, significantly improving the accuracy and efficiency of health monitoring. Random Forest (RF) performs exceptionally well in motion function assessment. Catherine’s research team [61] used six Shimmer2 sensors to collect upper limb motion acceleration data, extracted extreme values, root mean square, and other features through RF, and constructed the Fugl-Meyer Assessment model, with a coefficient of determination of 0.86 and root mean square error of 3.99 points, showing significant consistency with clinical scores. The Shimmer2 sensor is a wearable inertial measurement unit that operates based on micro-electromechanical systems technology. It integrates triaxial accelerometers and gyroscopes to capture linear acceleration and angular velocity, respectively. The raw kinematic data provide a high-dimensional representation of limb segment movement, which serves as the input for machine learning models to derive quantitative movement metrics. In the monitoring of Achilles tendon load, the LASSO regression model processed inertial measurement unit data, and the average absolute percentage error of the personalized model for Achilles tendon load prediction was as low as 8.35% [62]. The SVM demonstrated advantages in gesture recognition, achieving a classification accuracy of 99.37% for muscle electrical signals. CNN excels in extracting local features, with one-dimensional CNN achieving an accuracy of 98.3% for the recognition of blinking, up-down, and left-right eye movements in eye electrical signals, and real-time performance superior to traditional algorithms [63]. The LSTM network decoded complex hand movements by capturing temporal dependencies and achieved an accuracy of over 92%. The hybrid model further enhanced performance, such as the CNN-BiLSTM-attention mechanism model processing photoplethysmography signals, with the average absolute error of systolic blood pressure and diastolic blood pressure reaching 1.88 and 1.34, respectively, without the need for additional electrocardiogram signal input [64]. Self-supervised contrastive learning trains models using unlabeled data to address the high cost of medical data annotation. Jacobsen et al. [65] adopted a ResNet architecture with 24 residual blocks, mapping physiological parameter time series to a 128-dimensional feature space, achieving a sensitivity of 79.7% for detecting severe clinical complications in blood malignancy treatment and providing early warning 48 h in advance. Transfer learning reduces the need for new user data through meta-learning and quickly adapts to individual differences, thereby significantly improving the generalization ability of the model in gesture recognition.

The combination of flexible sensors and ML enables the quantitative assessment of the rehabilitation process. In the recovery of upper limb function in stroke patients, wearable devices collect data from eight functional movement tasks, and the RF model accurately estimates the FMA and functional ability scale scores, supporting personalized rehabilitation plan adjustments [61]. For post-operative rehabilitation of Achilles tendon injuries, the IMU sensor combined with LASSO regression predicts the peak load of the Achilles tendon and walking speed in real time, with the MAPE of the generalized model for walking speed prediction being ≤10.8% [62]. In monitoring elderly Tai Chi training, 13 IMU sensors capture full-body movements, and the RF model distinguishes the micro F1 score of six movements, reaching 90.05%, and realizes the assessment of proficiency levels [66]. In the cardiovascular monitoring field, the thin and soft wearable system uses snake-shaped flexible electrodes and piezoelectric materials, combined with the extreme gradient boosting algorithm, to achieve continuous monitoring of arterial blood pressure, with a systolic blood pressure error of −0.05 ± 4.61 mmHg, reaching the BHS standard A-level accuracy [67]. In diabetes management, sweat sensors detect sweat glucose through glucose oxidase-modified electrodes, and ML is correlated with blood glucose levels, thereby reducing skin temperature interference. The cuffless blood pressure monitoring system is based on PPG signals, and the deep learning model in the mixed model was validated in 2064 patient data, achieving high accuracy and providing a non-invasive solution for hypertension management [64]. During the COVID-19 pandemic, the Everion armband sensor continuously collected data such as heart rate and blood oxygen levels. The bio-life index generated by the Biovitals analysis engine showed a significant linear correlation with the viral load, with a sensitivity of 94.1% for predicting clinical deterioration events and an average 21-h early warning [68]. In the monitoring of children after appendectomy, the Fitbit device recorded data such as heart rate and activity level, and the balanced random forest classifier predicted infectious complications two days in advance, with a detection sensitivity of 83% [69]. For Friedreich’s ataxia, a motion capture kit with 17 IMUs combined with Gaussian process regression predicted the SARA score of disease progression with a coefficient of determination (R^2^) of 0.80, improving the accuracy by 1.7–4 times compared with traditional scales [70]. In the assessment of upper limb function in patients with Huntington’s chorea, the PAMSys sensor collected motion data, and the elastic net regularized logistic regression distinguished the HD, prodromal HD, and healthy control groups, with a balanced accuracy rate of 67% [71]. In the monitoring of X-linked dystonia-Parkinsonism, the Shimmer3 sensor combined with RF algorithms achieved a muscle dystonia detection accuracy of 0.94 for the hand pronation/supination task [72].

Despite these advancements, this field still faces several challenges. Signal interference is a particularly prominent problem in dynamic environments. Movement and temperature changes can affect the signal quality, and further optimization of the filtering algorithms is required. The comfort and biocompatibility of long-term wear still need to be improved. Although the moisture vapor transmission rate (MVTR) of breathable medical tape reaches 40.28 g/m^2^/h, the skin irritation problem has not been completely resolved [73]. However, the efficiency of multimodal data fusion is inadequate. The integration of physiological signals, motion data, and environmental parameters requires more efficient algorithms [57]. In addition, most systems lack large-scale clinical validation, and standardization and clinical translation remain important topics.

**Figure 8:**
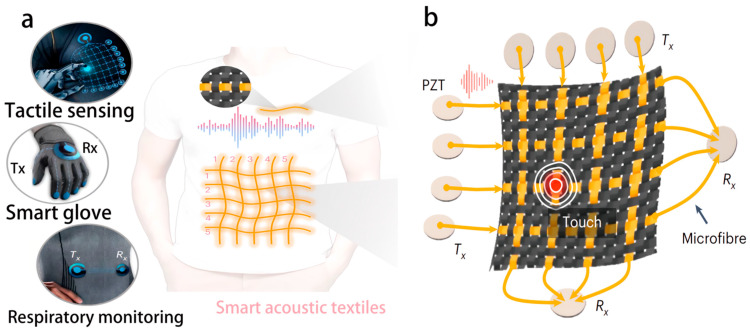
Flexible electronic technology and ML integration represent the technological evolution direction of wearable health monitoring. (**a**) The concept of the proposed intelligent acoustic textiles. The sound waves emitted and received by the piezoelectric ceramic (PZT) transducers will propagate along the micro-fiber waveguides woven into the textiles, completing various wearable sensing and interaction tasks. (**b**) Schematic diagram of the tactile sensing receiving array arranged in a single-input multiple-output pattern along the warp and weft lines. R3 can determine which Tx the sound wave comes from based on the frequency assigned for transmission and reception and can detect the coordinates of the touched area. Figure reproduced with permission from ref. [75]. Copyright from 2025, Springer Nature Limited.

In the technological evolution of wearable health monitoring systems, three core directions are driving the improvement of their performance and practicality. First, adaptive flexible materials, such as a base with thermal switching characteristics, adhere to the skin temperature and can be easily removed at low temperatures [58]. Second, lightweight algorithms such as the DeepECG-Net model with a latency of less than 50 ms when deployed on Raspberry Pi 4B, supporting real-time monitoring [74]. Third, human–computer interaction scenarios, such as intelligent acoustic textiles that achieve respiratory monitoring and gesture recognition through glass microfiber acoustic waveguides (Figure 8) [75], with a speech recognition system achieving an accuracy of 99.8% in noisy environments [9]. With the deep integration of flexible electronics and ML, wearable health monitoring systems will achieve a closed loop from “monitoring” to “prediction–intervention”, providing core technical support for personalized medicine and remote health management.

### 3.2. Intelligent Control of Soft Robots

Flexible electronic materials, as key foundational materials for soft robots, directly determine the motion flexibility of a robot’s body and its dynamic adaptability in complex environments. Dielectric elastomers, which are artificial muscle materials, form deformable capacitor structures through pre-stretching and carbon grease electrodes. Under a voltage drive, they achieved thickness changes and area expansion, providing a jet propulsion force for underwater soft squid robots. Combined with a magnetic bias mechanism, the total weight of the robot was only 126 g, enabling flexible movement in underwater environments [76]. Liquid metals, owing to their extremely high tensile strength and conductivity, are ideal choices for flexible circuits. Circuits fabricated through mask printing technology can integrate components, such as temperature sensors and accelerometers, meeting the signal transmission requirements of the robot under complex deformations [77]. The combination of carbon-based materials and flexible substrates further expands the application scenarios. For example, a strain sensor made by embedding carbon nanotubes in Ecoflex has a stretching rate of 98.5% and a response time of only 60 ms, capable of accurately capturing the subtle deformations of the robot [78]. Additionally, the combination of biocompatible materials such as chitosan and Fe_3_O_4_ nanoparticles enables a cross-shaped magnetic medical robot to achieve programmable deformation under a magnetic field drive, with a maximum displacement of 6.42 mm, which is suitable for minimally invasive surgical environments inside the body [79].

**Figure 9:**
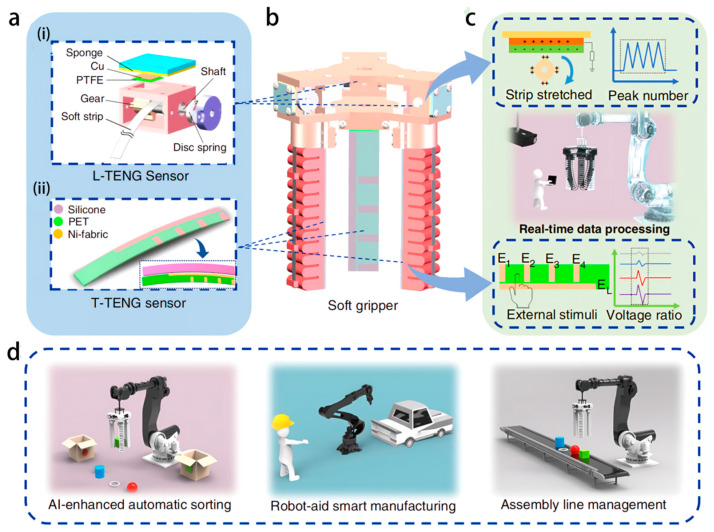
The structural diagram of the TENG used for soft robots. (**a**) The fabricated TENG sensor and its basic structure. (**i**) The length TENG (L-TENG) sensor and (**ii**) the tactile TENG (T-TENG) sensor. (**b**) The soft gripper integrated with the TENG sensor. (**c**) The intelligent sensing data processing strategy. E1 to E4 and EL represent the electrodes in the T-TENG sensor. (**d**) The digital twin application is based on the Internet of Things artificial intelligence sensing system. Figure reproduced with permission from ref. [80]. Copyright from 2020, Springer Nature Limited.

The integration of flexible sensors and actuators forms a perception-driven closed-loop system for soft robots, providing real-time data support for intelligent control. For tactile sensing, as illustrated in Figure 9, a triboelectric nanogenerator (TENG) detects the contact position, area, and sliding state by combining patterned Ni fabric electrodes with a silicone rubber layer. Distributed electrodes eliminate environmental interference through voltage ratio analysis, and in combination with the gear pulse signal of the length TENG (L-TENG), the soft grasping system can continuously detect the stretching length and bending angle, with a minimum resolution of 5 mm [80]. Pressure and strain sensing rely on the piezoresistive properties of carbon-based materials. The fully printed multimodal sensor array uses silver nanowires and carbon nanotube ink to fabricate tactile sensors that can detect pressures ranging from 0 to 500 Pa. Chemical sensors can identify TNT, organophosphorus pesticides, and even SARS-CoV-2 and achieve stable conductivity under 100% strain through a paper-cutting structure [81]. In terms of actuators, pneumatic-driven PneuNet bending actuators, rope-driven fiber-reinforced actuators, and photo-thermal-responsive liquid crystals all achieve complex movements, such as bending and twisting, through air pressure regulation, tendon contraction, and light stimulation. The tail of a biomimetic catfish robot, based on the fin effect, is 3D-printed using Extrudr FLEX medium material, achieving a maximum speed of 6 cm/s at a 1–2.5 Hz oscillation frequency, simulating the swimming characteristics of real fish [82,83,84].

ML overcomes the control challenges caused by nonlinear dynamics and high degrees of freedom of soft robots by analyzing sensor data and optimizing control strategies. Reinforcement learning performs exceptionally well in motion control, and the Q-learning algorithm defines the state as the past six action sequences and the action as the voltage output. After 25 training rounds, the swimming speed of the soft squid robot increased from 11 mm/s to 21 mm/s, and the action sequence converged from disorder to a periodic pattern [76]. The PPO algorithm demonstrated advantages in the target navigation task of the bionic catfish robot, with an accuracy rate of 95% for the robot to swim towards the target after 50,000 steps and the ability to switch between two targets, effectively overcoming the modeling difficulties of fluid–structure interaction [82]. Supervised learning plays a core role in environmental perception, and SVM combined with principal component analysis processes 15-channel TENG sensor data to achieve the recognition of 16 types of objects, with an accuracy rate of 98.1%. The recognition rate for similar spherical objects such as baseballs and tennis balls reached 95.0% [80]. The random forest algorithm accurately classifies five states with a 90.17% accuracy rate through the analysis of multimodal signals of the electronic ion skin (E-skin), providing precise environmental interaction feedback for the robot [85]. The model-free control framework further breaks through the limitations of traditional modeling, and the reservoir pool calculation trains the system’s state transition rules through random input signals, enabling complex trajectory tracking without the need for precise mechanical models, with the root mean square error of circular and “8”-shaped periodic trajectories approaching zero under weak interference [86]. The hybrid hierarchical learning framework decomposes tasks into sub-modules, combines behavior cloning and proximal policy optimization (PPO), and enables the robot to achieve a success rate of over 95% in tasks in seven complex scenarios with strong robustness to sensor noise [87].

In specific application scenarios, the combination of ML and flexible electronics demonstrates significant functional advantages, driving soft robots towards practicality. In the medical field, implantable soft robots achieve bladder volume monitoring and automatic electrical stimulation through a combination of electronic skin and PNIPAM hydrogel artificial muscles, with wireless radio frequency (RF) energy transmission supporting battery-free operation and a pressure monitoring sensitivity of 26.7 kHz·kPa^−1^, providing possibilities for precise medical intervention [77]. The snake-shaped bionic robot is made by 4D printing using magnetic-responsive intelligent ink, achieving segmented movement through 3T pulse magnetic fields, with a fluctuating swimming speed of 51.159 mm/s, and is capable of completing targeted drug delivery in vascular models [84]. In the industrial and detection fields, soft fluid robots use high-order differences in pressure-time curves to identify object size, stiffness, and maturity, achieving closed-loop control without additional sensors, suitable for fruit picking and automatic sorting [88], whereas the M-Boat robot based on fully printed flexible electronics uses an AI search algorithm combined with chemical sensor data to autonomously track dangerous compound sources, optimizing motion efficiency and decision accuracy [81]. In the human–machine interaction field, the WalkON soft robot shorts use Kevlar fiber tendons for driving, based on inertial measurement units (IMUs) to collect gait data, extracting gait phases through a high-order controller, reducing the metabolic cost of elderly people’s walking on flat ground by 10.48%, while balancing assistance effect and movement naturalness [89]; The electronic ion skin (E-skin) combined with decision tree algorithms enables remote control of 6 gestures with a delay of less than 200 milliseconds, providing intuitive interaction methods for remote robot operation [81].

Despite significant progress, this field still faces several challenges. The strong nonlinearity and hysteresis of soft robots make it difficult to achieve precise modeling, and traditional control methods cannot cope with parameter drift in dynamic environments. For instance, the pressure-deformation relationship of pneumatic actuators is affected by temperature and material aging, and continuous calibration using ML is required [90,91]. The insufficient efficiency of multimodal data fusion limits the accuracy of perception. The temporal-spatial synchronization and feature extraction of multi-source signals, such as tactile, strain, and temperature signals, have not yet formed a unified framework. For example, the accuracy of the electronic ion skin in identifying mixed states is approximately 1.6% lower than that in a single state [85]. However, long-term stability and reliability remain bottlenecks for practical applications. The dielectric elastomers of underwater robots are prone to fatigue under a high-frequency voltage drive, and the signal drift rate of carbon-based sensors in humid environments reaches 5–8% [76,78]. Moreover, a contradiction between unropedization and miniaturization is prominent. The energy supply and data transmission of micro-robots are limited compared to ultrasonic-driven micro-robots, which, although achieving a 90% target navigation success rate, still have a limited endurance time owing to the stability of microbubble self-organization [92].

**Figure 10:**
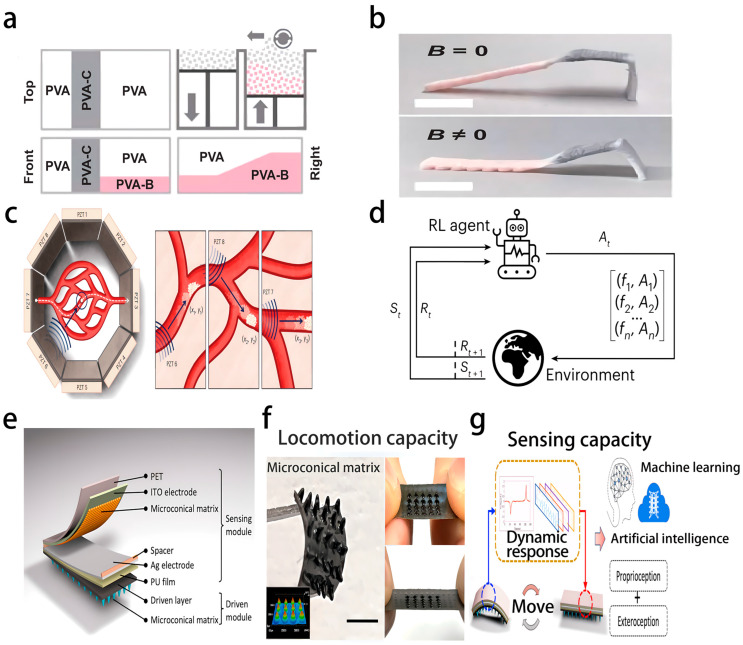
Future Development Directions of Soft Robots. (**a**,**b**) Multi-stimulus responsive soft robots based on functional gradient additive manufacturing technology (FGMM). (**a**) Schematic diagram of the multi-stimulus responsive FGMM-based base design for unencumbered soft robots. (**b**) The ratchet motion strategy of the soft robot. Figure reproduced with permission from ref. [94]. Copyright from 2024, International Journal of Extreme Manufacturing. (**c**,**d**) An autonomous ultrasonic-driven soft micro-robot. (**c**) Schematic diagram of the experimental device, which has an artificial blood vessel channel, with eight piezoelectric ceramics (PZTs) arranged in an octagonal structure around the channel. (**d**) Hierarchical representation of multiple possible scenarios to enhance the success rate of the algorithm. Figure reproduced with permission from ref. [92]. Copyright from 2025, Springer Nature Limited. (**e**–**g**) Overview of magnetic soft millimeter robots. (**e**) Schematic diagram of enhancing the motion ability of millimeter robots. The illustration shows the 3D surface profilometer results of the robot. (**f**) Micro-conical matrix in the millimeter robot (scale: 10 mm). (**g**) Sensing of the millimeter robot combined with ML. Figure reproduced with permission from ref. [96]. Copyright from 2025, Springer Nature Limited.

Future developments will focus on three major directions: material innovation, algorithm optimization, and scene expansion. In terms of materials, self-healing ionic gels achieve biological-like learning and memory functions through cation-π interactions. After five groups of 100-s stimulation training, the memory can be retained for five days, providing a material foundation for autonomous decision-making for robots [93]. 4D-printed functional gradient hydrogel networks combined with humidity and magnetic responses have a 72-h degradation rate of 96.6%, promoting the development of environmentally friendly soft robots (Figure 10a,b) [94]. At the algorithm level, model-based reinforcement learning (such as Dreamer v.3) increases the training efficiency by 50 times, enabling ultrasonic-driven micro-robots (Figure 10c) to adapt and achieve a 90% success rate within 30 min in new environments (Figure 10d) [92]. The combination of capsule neural networks (CapsNet) and improved spring search algorithm (MSSA) enables the gesture recognition accuracy of spider-like medical robots to reach 99.31%, with a processing time of only 0.5 s [95]. In scene applications, the medical field will achieve a closed loop from diagnosis to treatment, such as magnetic soft micro robots (Figure 10e,f) that use the time perception network (TPN) in ML (Figure 10g) to recognize five types of terrain, with an accuracy rate of 96.7%, and can complete vascular detection within a 3 mm channel [96]. In the industrial field, it will develop towards cluster collaboration, with snake-shaped robots improving speed by 3.8–4.1 times through the cooperation of fast and slow robots in pushing and pulling and performing coordinated operations in complex environments [84].

The collaborative development of ML and flexible electronics has driven soft robots from laboratory to practical applications. Through the deep integration of materials, algorithms, and scenes, core issues, such as nonlinear control and environmental adaptability, are gradually being solved. In the future, with the improvement of biocompatibility and breakthroughs in unroped technology, soft robots will play an irreplaceable role in minimally invasive surgery, hazardous environment detection, and human–machine collaboration, becoming the key carrier for the practical application of flexible intelligent systems.

### 3.3. Performance Optimization of Self-Powered Devices

Through the high adaptability of flexible materials and the precise regulation of intelligent algorithms, the coordinated development of energy collection efficiency improvement, signal analysis accuracy optimization, and function scenario expansion has been achieved. Flexible electronic materials, as the core carrier of self-powered devices, directly determine the basic performance of energy collection and sensing. In the frictional electrostatic material system, the PET friction layer and PVC substrate form an intelligent footpad through screen printing of silver electrodes, which realizes self-powered operation through contact electrification and electrostatic induction coupling with a maximum output power of 800.84 μW, capable of driving the operation of IoT sensors. The unique electrode “identity” design only requires two output electrodes to distinguish signals from multiple regions, significantly reducing system complexity [97]. In the piezoelectric material aspect, PVDF and its copolymers are regulated by electrospinning or 3D printing to control the β phase content, with the piezoelectric coefficient reaching −38 pC/N, while the output voltage of 1–3 type PZT nanowires/PDMS composite materials can reach 209 V, which can efficiently collect mechanical energy from human movement [98]. Biocompatible materials expand medical scenarios, such as the integration of a degradable PLLA-PTMC copolymer base with an Mg-FeMn primary battery to provide a self-powered supply for peripheral nerve interfaces, which can operate stably in a 37 °C physiological environment for 5–8 weeks before natural degradation, avoiding secondary surgeries [99]. Hydrogel materials exhibit excellent flexibility and conductivity, with a double-network polyacrylamide/sodium alginate hydrogel achieving a maximum elongation of 3000% and a gauge factor of 11.41, further enhancing its adaptability in strain sensing [100].

Self-powered technology builds a continuous power supply system through multiple mechanisms of energy collection, thereby laying the foundation for the long-term operation of devices. TENG is one of the mainstream solutions, in which electrospun PVDF-BaTiO_3_ nanofiber membranes are combined with nickel fabric electrodes to utilize a high specific surface area to enhance the frictional electrostatic performance. Some devices can output voltages up to 2.1 kV, capable of collecting mechanical energy from breathing and movement [101]. The ATH-Ring TENG, based on a silicone pyramid structure and a thermosensitive PVDF film, integrates the self-powered perception of finger bending and temperature and also supports vibration and thermal tactile feedback [102]. The piezoelectric generator (PENG) focuses on capturing minute energy, with flexible PZT/mica films achieving an energy density of 42 mW/cm^3^ through transfer technology, capable of collecting weak mechanical energy, such as heartbeats [98]. Biomechanical energy collection expands new paths, such as enzyme-based fuel cells (EBFCs), which use glucose and lactic acid in sweat as fuel and achieve μW to mW level power output through immobilized glucose oxidase (GOx), suitable for long-term power supply in wearable scenarios [103]. Additionally, flexible thermoelectric generators (f-TEGs) have a power density of 3.5 μW/cm^2^ and can generate electricity using the temperature difference between the human body and the environment. Hybrid energy systems, such as a solar-frictional energy hybrid system that combines multiple energy sources to reduce the limitations of a single mechanism, further enhance stability [104].

**Figure 11:**
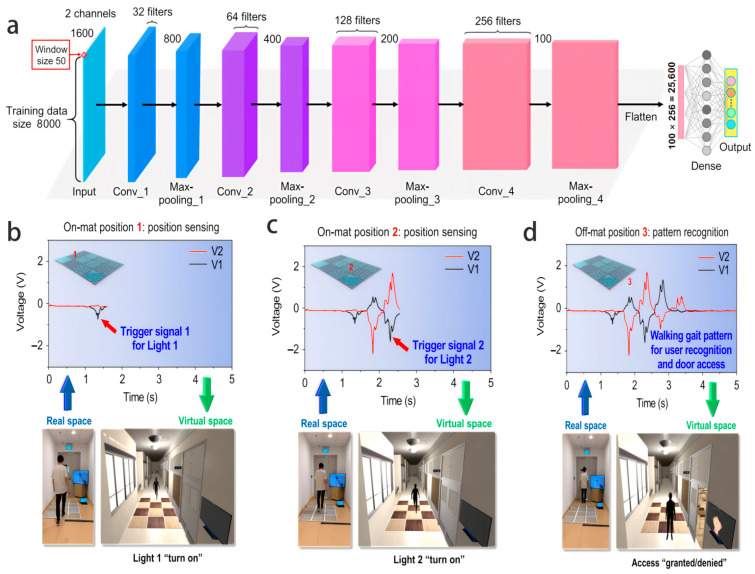
Self-powered intelligent footpad monitoring system integrating deep learning for data analysis. (**a**) Detailed structure of the convolutional neural network (CNN) training model. (**b**–**d**) Demonstrations of different stages of real-time location perception and individual identification, where a person walks in the real space while his digital twin is controlled to walk correspondingly in the virtual space: (**b**) At position 1, the first negative peak is detected and used to turn on light 1; (**c**) At position 2, the third negative peak is detected and used to turn on light 2; (**d**) At position 3, the complete walking signal is detected and analyzed in the CNN model for individual prediction and access control. Figure reproduced with permission from ref. [97]. Copyright from 2020, Springer Nature Limited.

ML achieves a comprehensive performance improvement of self-powered devices through the analysis of sensing signals, optimization of material parameters, and regulation of energy distribution. In signal processing and pattern recognition, CNN demonstrates strong feature extraction capabilities (Figure 11a), with an identification accuracy of 96% for 10 users when processing gait signals of the intelligent footpad and a cross-state recognition accuracy of 89.17% (Figure 11b–d) [97]. When analyzing neural signals for degradable nerve interfaces, the accuracy of identifying the swing phase of gait was 77%, the support phase was 85%, and the accuracy of the early detection of neural tumors was 94% [99]. The SVM performs exceptionally well in multimodal recognition. When processing the tactile signals of ATH-Ring, the accuracy rate of recognizing 14 American Sign Language gestures reached 99.82%, and the recognition rate for eight daily items exceeded 96% [101]. In the classification of laryngeal movement signals, the multi-class SVC algorithm increased the accuracy rate of speech command recognition to 94.68%, assisting patients with vocal cord dysfunction in vocalization [105]. Unsupervised learning and optimization algorithms accelerate the material and structure design. K-means clustering divided the five deformation modes of the Kigali metamaterial into five categories, and a tandem deep neural network (T-DNN) was used to achieve the reverse design. The R^2^ value for predicting incision parameters based on target deformation reached 0.96–0.98 [106]. Bayesian optimization iteratively optimized the concentrations of the seven components of the hydrogel, and the random forest model accurately predicted its resistivity and elongation rate, significantly improving the efficiency of material preparation [100]. Neural morphological computing promotes a low-power design. The asynchronous sensing computing chip “Speck” adopts an event-driven architecture with a static power consumption of only 0.42 mW. Combined with the attention mechanism, it reduces pulse counting by more than 60% while increasing the accuracy rate of gesture recognition by 9.0% [107].

**Figure 12:**
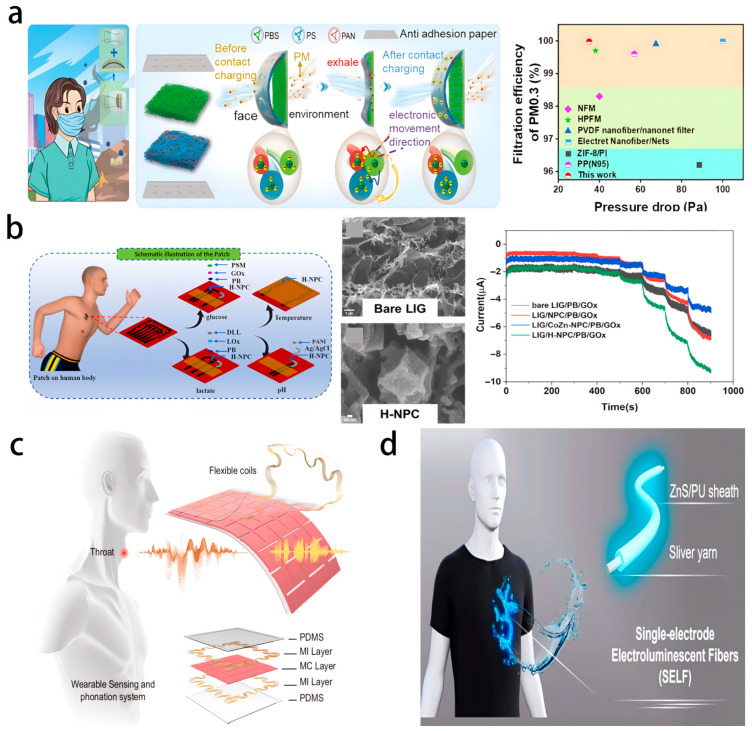
The intelligent self-powered system integrating ML and flexible electronics precisely interprets signals. (**a**) The intelligent mask of TENG combined with the ML algorithm captures breathing patterns. Figure reproduced with permission from ref. [101]. Copyright from 2025, Springer Nature Limited. (**b**) EBFCs combined with ML monitors data while maintaining the self-stabilizing power supply of the device. Figure reproduced with permission from ref. [103]. Copyright from 2024, Springer Nature Limited. (**c**) The wearable sensing and speaking system attached to the throat combines ML to assist patients with vocal cord dysfunction in communication. Figure reproduced with permission from ref. [105]. Copyright from 2024, Springer Nature Limited. (**d**) ML assists flexible intelligent textiles in dynamically adjusting signals. Figure reproduced with permission from ref. [108]. Copyright from 2025, Springer Nature Limited.

The intelligent self-powered system, combined with advanced algorithms, enables the real-time and precise analysis of physiological signals. For instance, the intelligent mask of a TENG can capture breathing patterns and accurately identify various states such as normal breathing and deep breathing (Figure 12a) [101]. Wearable devices based on enzyme biofuel cells (EBFCs) utilize glucose and lactic acid in sweat as fuel and combine ML to process multiparameter biological signals. This not only enables real-time monitoring of blood sugar and lactate levels but also dynamically optimizes enzyme catalytic conditions to maintain a stable power supply for the device (Figure 12b) [103]. Self-powered flexible devices provide a new paradigm for seamless interaction. A screen-printed carbon nanotube/polyurethane (CNT/PU) triboelectric textile sensor with a high permeability of up to 88 mm/s, combined with deep learning algorithms to process capacitance signals, significantly improves the accuracy and robustness of gesture control [108]. Multi-modal interaction devices such as ATH-Ring, which integrate triboelectric and thermoelectric sensing, process finger bending, temperature, and tactile signals through ML, not only support high-precision (>96%) daily item recognition and American Sign Language translation (99.82% accuracy), but also enable cross-space tactile feedback in the metaverse [102]. Self-powered technologies provide innovative solutions for medical interventions. The soft magnetic elastic self-powered laryngeal sensing actuation system utilizes PCA to extract laryngeal movement features and drive the actuation component to output speech, ensuring stable operation even in the presence of user movement or sweating, providing an effective auxiliary means of communication for patients with vocal cord dysfunction (Figure 12c) [105]. The biodegradable neural interface (Bioreactor) integrates self-powered units with neural signal acquisition, using CNN to analyze neural signals, not only accurately identifying gait phases with high accuracy but also early predicting the recovery status of nerve damage and detecting neuromas, optimizing the treatment strategy for peripheral nerve injuries [99]. Furthermore, self-powered devices provide core perception capabilities in intelligent environments. The intelligent footpad with a unique electrode “identity” design requires only two output electrodes to achieve multi-region signal distinction and user identity verification, supporting real-time location perception of buildings, automatic lighting control, and intelligent access control [97]. Innovative sensory interaction fibers can convert mechanical energy into light and electrical signals, and through integrated computer vision, dynamically adjust the color mode of light, greatly enriching the application scenarios of intelligent textiles (Figure 12d) [108].

However, the current field still faces several challenges. The balance between the energy conversion efficiency and stability is the core issue. Frictional electrical devices are prone to signal drift under high-frequency dynamic conditions. The fatigue life of piezoelectric materials is significantly affected by the temperature and humidity. Some TENGs experience 15–20% signal attenuation in humid environments. The insufficient temporal-spatial synchronicity of multimodal signal fusion is a problem. For instance, the time delay between tactile and temperature signals may lead to a 3–5% decrease in the object recognition accuracy of ATH-Ring [102]. The contradiction between biocompatibility and long-term stability was prominent. The GOx activity of the enzyme biofuel cell decreased by approximately 30% after one week, affecting the continuity of the power supply [103]. In addition, the generalization ability of ML models is limited. The SVM accuracy drops by 8–10% when cross-user gesture recognition is performed, and transfer learning is required to reduce the impact of individual differences [8].

Therefore, advanced materials that are efficient in energy conversion, have excellent environmental stability, and are biocompatible should be developed. Key directions include exploring the combination of self-healing hydrogels and dynamic covalent networks and using reversible cross-linking mechanisms to enable self-repair of frictional electrical materials after fatigue damage [100]. For long-term signal stability in high-humidity environments, a multi-level encapsulation and compensation strategy is proposed. This includes developing self-healing hydrophobic polymers to autonomously repair micro-cracks, integrating 2D hydrophobic materials (e.g., fluorinated graphene) into triboelectric layers to maintain charge transfer efficiency, and adopting hermetic sealing with inert gas or hydrophobic gel fillers. Coupled with in situ impedance monitoring and adaptive signal compensation algorithms, this integrated approach aims to sustainably mitigate the 15–20% signal attenuation under varying humidity conditions, ensuring reliable long-term operation of self-powered sensors.

Developing biocatalysts with a longer active retention period to solve the problem of glucose oxidase (GOx) activity decreasing by approximately 30% after wearing for one week in EBFCs, ensuring a continuous power supply [103]. Enhancing the generalization ability, real-time performance, and energy efficiency of algorithms is crucial. Integrating federated learning with edge computing architectures, while protecting user privacy, uses distributed training models to process sensor data from multiple devices, improve model robustness, and reduce the decline in recognition accuracy due to individual differences [107]. To address the issue of SVM accuracy dropping by 8–10% in cross-user gesture recognition, a hybrid approach combining domain adaptation and ensemble learning strategies is proposed. Specifically, a domain-adversarial neural network (DANN) can be integrated with SVM to minimize the distribution discrepancy between training and testing users, thereby enhancing model generalization. Additionally, an ensemble of SVMs trained on diverse user subsets can be employed, weighted by user similarity metrics derived from kinematic features, to reduce the impact of individual variability. This approach not only mitigates accuracy degradation but also maintains low computational overhead, making it suitable for real-time, embedded self-powered systems. Furthermore, incorporating incremental learning mechanisms allows the model to adapt continuously to new users without retraining from scratch, thus supporting long-term deployment in practical applications. Developing lightweight neural network models and dedicated neural morphological computing hardware to further reduce energy consumption in signal processing and pattern recognition. Exploring reinforcement learning algorithms to dynamically allocate and manage energy in hybrid energy collection systems, maximizing the overall efficiency and stability of the system [104]. Promoting the penetration of self-powered technologies into deeper and more complex scenarios. Implantable self-powered devices for closed-loop therapy, such as an integrated system that integrates real-time neural signal analysis and on-demand drug delivery functions, can achieve a closed loop in diagnosis and treatment [99]. Expanding the reliable operation capability of devices in extreme or complex biological environments. Deepening multimodal perception fusion technology to solve the problem of temporal-spatial synchronicity, achieving more natural and immersive human–computer interaction [100]. Establishing a unified and objective performance evaluation standard is crucial for promoting technology. There is an urgent need to develop core indicator testing specifications and standard procedures for energy conversion efficiency, long-term stability, biocompatibility, and signal quality of different types of self-powered devices, providing reliable comparison benchmarks and industrialization bases for the academic and industrial communities.

### 3.4. Intelligent Perception of Epidermal Electronic Systems

ML plays a central role as an intelligent core in smart-epidermal electronic systems. Its powerful capability lies in being able to efficiently parse multi-dimensional and multi-modal physiological and physical signals from the flexible sensor array, converting raw, high-noise biological, electrical, mechanical, chemical, etc., data streams into high-level information with clear clinical diagnostic value or human–computer interaction instructions. This process overcomes the limitations of traditional signal processing methods based on thresholds or simple filtering, significantly enhancing the practicality and intelligence level of the system. In a copper (Cu) electrode-based metal matrix material system, the epidermal electromyography tattoo patch encapsulated by polyimide (PI) has a thickness of less than 10 μm, a peel strength of over 1 N/cm^−1^, and a double-line wave interconnection structure that can withstand a 20% strain, enabling stable adhesion to complex curved areas such as the face and jaw, providing a reliable interface for electromyography (sEMG) signal collection [109]. The networked electrode formed by the combination of silver nanowires (Ag NW) and thermoplastic polyurethane (TPU) has a tensile strength of 130%, and it can withstand 800% strain, balancing conductivity and flexibility [110]. Carbon-based materials exhibit excellent breathability and transparency, with graphene electronic tattoos having a thickness of less than 1 μm, a water vapor permeability of 1748.09 g·m^−2^·d^−1^, and suitability for long-term epidermal monitoring [111]. An electronic nose (E-nose) composed of carbon nanotube sensor arrays can quickly respond to volatile metabolites during breathing, completing non-invasive breast cancer detection within 30 min [112]. Conductive polymers and hydrogels further expand biocompatibility, with PEDOT: PSS transferring graphene made into an ultra-adaptable skin electrode (PTG) with a conductivity of 4142 S/cm and an interface impedance of only 32 kΩ at 100 Hz, significantly reducing motion artifacts [113]. Carboxyethyl chitosan-polyacrylamide hydrogel (CTA hydrogel) achieves a maximum stretching rate of 1586%, with a conductivity of 0.62 S/m, and can parse micro-physiological signals such as pulse wave P/T/D waves [114].

The design of a smart epidermal sensor focuses on high sensitivity and low interference, thereby constructing a multidimensional signal collection network. In the field of tactile perception, the TRACE sensor with platinum (Pt) film deposited on the PDMS micro pyramid structure, formed by soft indentation processing into regular ring-shaped cracks, has a sensitivity of over 10^7^ Ω·kPa^−1^, a lag of only 2.99 ± 1.37%, and is capable of accurately capturing 5 Pa of tiny pressure, with stable performance after 50,000 cycles [115]. The Au-coated PDMS micro-column of the skin-electrode mechanical sensing structure (SEMS) responds to changes in contact area through buckling instability, with a sensitivity of 11.8 kPa^−1^ and a detection limit as low as 0.2 Pa, capable of distinguishing the subtle waveforms of fingertip pulse [116]. In terms of physiological signal monitoring, an ultrathin crystalline silicon strain gauge (SiNM) has a thickness of less than 8 μm, achieving 30% stretch through a grid and serpentine structure, with a double-axis strain sensitivity 20.8 times that of metal sensors, suitable for signal collection from dynamic areas such as the throat [117]. The mechanically interlocked silver nanowire/water gel mixed electrode interface has a toughness of 158.2 J·m^−2^, with a skin contact impedance superior to commercial Ag/AgCl electrodes, capable of high-fidelity recording of electromyography, electrocardiograms, etc., and biological signals [118]. Multi-modal integrated sensors further enhance the scene adaptability. The bone surface electronic system integrates metal foil strain gauges, NTC thermistors, and micro-LEDs, capable of real-time monitoring of bone strain (resolution 14.3 µε) and temperature (<10 mK) and providing light stimulation, operating wirelessly and battery-free via 13.56 MHz NFC [119]. Electronic textiles incorporate boron nitride nanoparticles and silver nanowires to form a 3D heat transfer network, providing EMI shielding (SE_t_ > 65 dB) and UV protection (UPF = 143.1). They can stably collect sEMG and ECG signals in complex environments [120].

**Figure 13:**
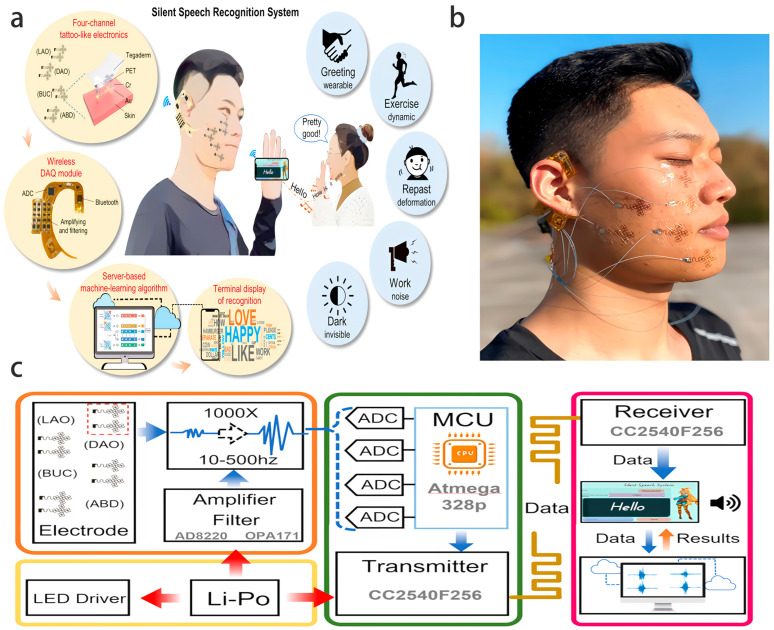
Design of an audible voice recognition system using electronic tattoos combined with ML. (**a**) Schematic diagram of the audible voice recognition system, including a four-channel electronic tattoo device, a wireless data acquisition module, a server-based ML, and a terminal display for the recognition results. (**b**) Photo of the participant wearing the audible voice recognition system. (**c**) Functional block diagram of the wireless data acquisition module. Figure reproduced with permission from ref. [121]. Copyright from 2021, Springer Nature Limited.

After being parsed by ML, the multi-source signals collected by the intelligent perception of epidermal electronic systems can convert the raw data into information with clinical or interactive value, breaking through the limitations of traditional signal processing. Linear discriminant analysis (LDA) demonstrated efficient feature extraction capabilities. When processing four-channel electromyography signals, the recognition accuracy for five action instructions reached 89.04%, and for six emotional instructions, it reached 92.33%, helping speech-impaired patients achieve silent communication [109]. Additionally, for the daily vocabulary recognition of tattoo electronic devices, combined with wavelet packet denoising and 15 frequency domain feature extractions, the average recognition accuracy of 110 words reached 92.64%, with a single-channel accuracy remaining at 42.27% (Figure 13) [121]. Deep learning models outstandingly perform complex signal analyses. The CNN processes the tactile images of the TRACE sensor, achieving a single-contact recognition accuracy of 94.3% ± 5.3% for 10 surface textures, which is significantly higher than the 68.3% ± 13.5% of the high-lag sensor [115]. Moreover, 3D CNN analyzes 8-channel silicon-based strain data, achieving a 5-fold cross-validation accuracy of 87.53% for 100 words and precisely locates the key signal areas through the relevant class activation map (R-CAM) [117]. Ensemble learning algorithms enhance the diagnostic reliability. The random forest model processes the breast cancer respiratory biopsy data from the electronic nose, achieving a diagnostic accuracy of 91% (AUC 0.99) and an 88.5 ± 12.1% accuracy rate for the leave-one-out cross-validation of molecular subtypes [112]. Furthermore, when used for breast cancer biomarker analysis, the integrated performance was optimal after balancing the data, with an accuracy of 83%, and showed good stability in cross-validation [122].

The smart epidermal electronic system integrates flexible sensing with ML, promoting noninvasive and precise diagnosis. The swallowing function assessment device adheres metal-based and carbon-based sensors to the neck to monitor electrical muscle signals and laryngeal movements during swallowing. SVM has an accuracy rate of approximately 96% in distinguishing swallowing and coughing activities, and the deep learning algorithm can achieve swallowing volume estimation and abnormal detection [123]. The bone surface electronic system wirelessly monitors bone strain and temperature, combined with a deep neural network (DeepLabCut) to analyze the gait of rats, providing quantitative data for fracture rehabilitation and osteoporosis assessment without interfering with normal movement [119]. ML significantly enhances the natural interactions and functional compensation capabilities of flexible electronic devices. The epidermal electronic device based on Joule heating strain isolation processes muscle electrical signals using the DenseNet100 model, with a 6-gesture recognition accuracy rate of 91.83%, superior to the 82.33% of commercial sensors, and suitable for prosthetic control scenarios [124]. The intelligent glove integrates 65 SEMS sensing units and uses big data analysis to identify object stiffness and shape, helping patients with tactile dysfunction to restore their perception [116]. The collaboration between flexible electronics and intelligent algorithms ensures signal reliability in complex environments. The TRACE sensor array achieves cardiovascular health assessment through pulse wave velocity (PWV) monitoring, with low-lag characteristics ensuring the stability of ML models [115], and electronic textiles can still interpret ECG P/QRS/T waves and EEG multiband signals under strong electromagnetic interference, providing high-quality data for daily health management [120].

Although ML-assisted flexible electronics have achieved significant breakthroughs in smart epidermal electronic systems, their large-scale application remains limited by the following core challenges. In terms of material stability, conductive polymers are prone to erosion by sweat during long-term wear, and the conductivity of PEDOT:PSS electrodes decrease by approximately 15% after 8 h [113]. The adhesion force of hydrogels fluctuates with humidity, and detachment may occur in high-humidity environments [114]. Motion artifacts remain the main obstacle in signal analysis, and skin deformation caused by facial muscle movement reduces the signal-to-noise ratio (SNR) of electrical muscle signals by 30–40%, affecting the accuracy of silent speech recognition [121]. During joint movement, the contact impedance of traditional electrodes can change by up to 200%, interfering with the continuous monitoring of electrocardiogram signals [110]. The insufficient generalization of algorithms limits cross-scenario applications, with the accuracy rate of gesture recognition models varying by 15–20% between different users and requiring reliance on transfer learning to reduce individual differences [124]. The sensitivity of breast cancer diagnosis models for early micro-diseases is only 68%, which is lower than the clinical requirements [112]. Moreover, the lack of a standardized system for clinical translation and the fact that most devices have not undergone long-term biocompatibility verification, such as the in vivo stability of the bone surface electronic system, has only been verified for up to 12 weeks [119].

**Figure 14:**
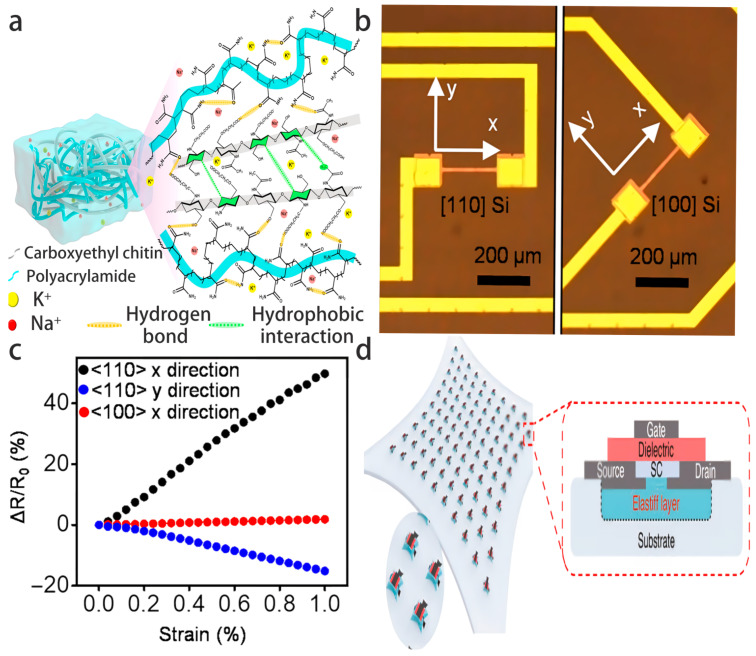
The flexible electronic technology assisted by ML drives the development of intelligent perception of epidermal electronic systems. (**a**) Schematic diagram of the intermolecular interactions in self-healing hydrogels. Even when stretched to a 1600% strain, it does not break and can be combined with ML for epidermal electronics. Figure reproduced with permission from ref. [114]. Copyright from 2023, Springer Nature Limited. (**b**) Schematic diagram of the deformation of flexible sensors caused by multiple sources of signals. The relationship among the three parameters (i.e., stimulation intensity, substrate hardness, and sensor output) in Figure (**c**) reflects the data regression assisted by deep learning, which is the algorithm optimization of ML-assisted epidermal electronic signals. Figure reproduced with permission from ref. [111]. Copyright from 2022, Springer Nature Limited. (**d**) Organic thin-film transistors are used in degradable epidermal electronic systems. Figure reproduced with permission from ref. [125]. Copyright from 2023, Springer Nature Limited.

The ML-assisted flexible electronic technology drives the coordinated development of smart epidermal electronic systems. Driven by the ML model, advanced flexible materials exhibit key performance. Self-healing hydrogels use dynamic covalent bonds to achieve performance recovery after damage, providing a solution to the aging problem of long-term wearable devices (Figure 14a) [114]. Interface-engineering-optimized nanocomposites significantly enhance the resistance to sweat erosion, with an anti-corrosion performance improvement of up to three times [118], thereby laying the physical foundation for reliable data collection. Algorithm optimization is the core breakthrough; the integration of federated learning and edge computing significantly improves real-time processing capabilities while protecting user privacy, and distributed training models reduce the accuracy difference in cross-user gesture recognition to within 5% [110]. The multimodal data fusion algorithm effectively integrates multi-source signals, such as electromyography, strain, and temperature from flexible sensors (Figure 14b), significantly enhancing the interaction robustness in complex environments (Figure 14c) [111]. With the empowerment of ML, application scenarios are penetrating deeply into clinical fields, and degradable epidermal electronic systems based on ML analysis achieve short-term postoperative monitoring and complete natural degradation after 8 weeks (Figure 14d) [125]. By drawing on the idea of plant epidermal electronic patterns, the developed system, combined with the real-time analysis capabilities of ML, is expected to be used for the continuous monitoring of body surface tumor markers [126]. It is necessary to increase the intensity of basic research and technology development, explore more application scenarios, and fully leverage the collaborative advantages of flexible electronics and ML.

Furthermore, the integration of ML extends beyond the intelligent perception of completed systems to the very fabrication process of flexible electronic devices themselves, ensuring performance from the outset. A prime example is the optimization of high-resolution manufacturing techniques like electrohydrodynamic-jet printing. As demonstrated by Abbasi Shirsavar et al., supervised ML models were trained on experimental datasets correlating key printing parameters—nozzle speed, voltage, and ink flow rate—with the resulting electrical conductivity of printed graphene electrodes [127]. This approach achieved prediction accuracies up to 83% for classifying electrode conductivity, effectively transitioning the manufacturing paradigm from empirical trial-and-error to a predictive, data-driven process. Such ML-assisted real-time process control minimizes material waste, enhances production yield, and guarantees the consistent quality of flexible components like epidermal sensors before their deployment. This underscores that ML serves as a pivotal tool not only in interpreting signals from flexible electronics but also in intelligently governing their creation, thereby reinforcing the closed-loop synergy between advanced manufacturing and reliable system performance in the flexible electronics ecosystem.

Currently, ML has become a key means to solve complex problems in the field of flexible electronics, and its combination with flexible electronics not only heralds the imminent profound transformation of flexible intelligent systems but also will inject new impetus into human life and industrial development. The transformative potential of ML in flexible electronics is most concretely realized in its practical applications. As summarized in Table 3, this synergy is driving innovation across four key domains.

### 3.5. Cross-Domain Transferable Methodologies: Bridging Industrial ML to Biosensing Paradigms

While the core of this review is anchored in biosensing applications, the underlying ML paradigms—particularly in sensor signal processing, anomaly detection, and edge intelligence—often originate or find mature expression in non-biological domains such as industrial automation, network security, and advanced manufacturing. The inclusion of selected examples from these fields is not merely illustrative but underscores a fundamental methodological synergy: the challenges of extracting reliable information from noisy, high-dimensional, and dynamically varying signals are ubiquitous across sensing modalities. Consequently, algorithmic innovations developed in one domain can be rigorously adapted and optimized to address analogous bottlenecks in flexible biosensing, accelerating the development of robust and intelligent health monitoring systems.

A prime example is the transfer of PDAD frameworks. In industrial vision systems, deep learning models like YOLO and Transformer-based architectures are deployed to identify micron-scale defects amidst complex backgrounds [39,41]. The core technical challenge—distinguishing subtle, patterned anomalies from normal variations under variable lighting and material conditions—directly parallels the problem of detecting physiological anomalies in biomedical time-series data. For instance, the shallow feature fusion networks and attention mechanisms used to pinpoint a 0.1 mm chip defect [38] are methodologically analogous to the architectures needed to identify arrhythmic heartbeats in an electroencephalogram (EEG) or epileptic spikes in an EEG from flexible epidermal sensors. The industrial domain provides a testbed for developing highly sensitive, low false-positive detection algorithms that, when retrained on annotated physiological datasets, can significantly enhance the early diagnosis of pathological states from wearable biosignals.

Similarly, principles from DCEC in resource-constrained industrial IoT and telecommunications [46,51] are directly applicable to wearable and implantable biosensors. The constraints are analogous: limited power budget, minimal latency tolerance, and the need for efficient data transmission or local processing. Techniques such as adaptive downsampling based on signal entropy, wavelet-based compression preserving clinical features, and lightweight model deployment developed for monitoring industrial equipment or plasma states [46] provide a blueprint for processing continuous streams of physiological data at the edge. This enables real-time health alerts and long-term monitoring without burdening cloud infrastructure or device battery life, a critical requirement for chronic disease management and personalized medicine.

Furthermore, multimodal fusion and robust feature extraction strategies pioneered in autonomous systems and cybersecurity offer valuable templates for biosensing. The fusion of inertial measurement unit data with biochemical sensor readings, or the integration of acoustic, mechanical, and electrical signals from the throat for speech recognition [9,33], faces similar challenges in alignment, dimensionality reduction, and cross-modal correlation modeling. Adopting and adapting these advanced fusion frameworks can improve the robustness of biosensing systems against motion artifacts and environmental interferences, which are major hurdles in ambulatory monitoring.

It is crucial to acknowledge that while the algorithmic cores are transferable, successful migration to the biosensing domain requires domain-specific adaptation. This includes retraining with physiologically relevant datasets, incorporating biophysical models into the learning process, and rigorously validating performance under real-world wearable conditions. The unique requirements of biocompatibility, long-term stability, and user comfort further shape the final implementation. Therefore, the examples cited from non-biological fields serve as a methodological repository and a source of proven architectural concepts, demonstrating that the ML-assisted flexible electronics ecosystem is inherently interdisciplinary. By leveraging these cross-pollinated advances, the biosensors community can avoid reinventing foundational signal processing wheels and instead focus innovation on solving the distinctive challenges at the biology-electronics interface, thereby accelerating the translation of flexible biosensing technologies from laboratory prototypes to clinically impactful and user-centric applications.

### 3.6. Toward Clinically Actionable Systems: Data Rigor and Evaluation Standards

The transition of ML-assisted flexible biosensors from compelling laboratory demonstrations to reliable, clinically actionable tools is critically dependent on the rigor of data validation and the relevance of evaluation metrics. While the preceding sections highlight remarkable algorithmic achievements—often reporting accuracy, sensitivity, or specificity exceeding 90% under controlled conditions—these performance figures must be critically appraised within the context of their experimental design. A significant gap persists, primarily defined by challenges in generalizability, label integrity, and clinical correlation, which are often under-addressed in proof-of-concept studies.

A foundational challenge is ensuring model generalization across diverse populations and over time. Many studies leverage data from small, homogeneous cohorts (e.g., 10–20 healthy subjects) collected within limited sessions, achieving high performance by learning subject-specific or session-specific patterns rather than robust physiological signatures. Consequently, models frequently degrade when applied to new individuals from different demographics or to the same individual on a different day due to factors like altered skin impedance, hydration, or daily physiological variation. This limitation directly undermines the promise of personalized, long-term health monitoring. Closely related is the issue of label quality, where the supervised learning paradigm’s efficacy is bounded by the accuracy of its ground truth. In wearable sensing, labels for activities, physiological states, or disease conditions are often derived from manual annotation, subjective self-report, or imperfectly synchronized reference devices, introducing noise and systematic bias that compromise the learning process and inflate perceived performance.

Furthermore, improper experimental design, particularly data splitting that fails to account for subject identity or temporal dependencies, can lead to data leakage. This creates an optimistic bias where models inadvertently memorize identifiers of the training set instead of generalizing, rendering reported metrics misleading for real-world deployment. Ultimately, the most pressing gap lies in connecting algorithmic performance to clinically meaningful endpoints. High accuracy in classifying a curated set of laboratory activities or recognizing gestures is a necessary but insufficient step. Clinical utility requires validation against accepted diagnostic gold standards, demonstration of improved patient outcomes, or integration into decision-making pathways that meet regulatory and clinical reliability thresholds. For instance, a cuffless blood pressure monitor must be validated according to protocols like ISO 81060-2 [128], not merely by correlation on a small, static dataset.

To bridge this demonstration-to-deployment gap, the field must adopt more stringent validation protocols. This includes enforcing strict subject-wise or session-wise data splitting to prevent leakage, and ideally, validating models on completely external, heterogeneous cohorts to stress-test generalizability. The collection and use of longitudinal, ambulatory datasets spanning days or weeks are essential to evaluate both sensor stability and model robustness against real-world variability. Ground truthing should be enhanced through multi-modal synchronization with high-fidelity clinical instruments. Reporting must move beyond aggregate accuracy to include detailed error analyses, confusion matrices, and metrics like F1-scores for imbalanced classes or Bland–Altman plots for continuous estimation, all benchmarked against established clinical or engineering standards.

It is, therefore, crucial to distinguish between a laboratory demonstration—which successfully validates a technological principle under optimized conditions—and a clinically actionable system. The former proves feasibility; the latter must demonstrate reliable, safe, and effective operation for the intended use case and population in real-world environments. Most work reviewed herein, including studies cited for their high performance, serves as vital pioneering research in the first category. The path forward necessitates a deliberate shift in research design, where studies are structured from the outset with clinical translation as a goal, incorporating the rigorous data practices and holistic evaluation standards discussed. Only through such a framework can ML-assisted flexible biosensors evolve from impressive prototypes into trustworthy instruments that genuinely augment healthcare diagnostics and monitoring.

## 4. Conclusions and Future Prospects

ML has constructed a complete chain of intelligent processing paradigms for flexible electronics, including “collection–processing–analysis–decision-making.” Four key information processing technologies (IPSS, MFE, PDAD, and DCEC) have achieved significant performance improvements in signal anti-interference, multi-source information fusion, micro-defect recognition, and efficient closed-loop processing of resource-constrained devices. Based on this, flexible electronics have achieved a leap from perception to decision-making in typical scenarios, such as wearable health monitoring, soft robots, self-powered devices, and epidermal electronic systems, significantly enhancing the practicality and scene adaptability of the system.

However, ML-assisted flexible electronics still face four challenges for large-scale applications: materials, processes, algorithms, and reliability. In terms of materials, the conductivity of conductive polymers decreases in sweat environments, the adhesion of hydrogels is affected by humidity, dielectric elastomers are prone to fatigue at high frequencies, and modeling of multi-physical field coupling behavior is difficult. In terms of the process, the yield of large-sized flexible arrays is low, the deformation interfaces are prone to failure, and the high cost restricts their promotion. In terms of algorithms, there are problems such as weak model generalization, difficult multi-modal fusion, and limited edge-computing power, resulting in a decrease in recognition accuracy. In terms of reliability, issues such as signal drift in humid environments, difficulty in monitoring hidden damage, and insufficient long-term stability of the system are prominent.

To address these challenges, multi-dimensional collaborative innovation is needed, and in terms of materials, self-healing mechanisms, hydrophobic coatings, and breathable graphene composites have been introduced, combined with molecular dynamics and ML to establish multi-physical field models. In terms of process, liquid metal printing, “island–bridge” structure design, and low-cost printing technologies are optimized to improve manufacturing yield and compatibility. In terms of algorithms and hardware collaboration, federated learning-edge computing architectures were adopted to enhance generalization and privacy protection, attention mechanisms were used to optimize fusion, and lightweight networks and knowledge distillation were used to reduce latency. To enhance reliability, adaptive calibration, hydrophobic encapsulation, shape-memory materials, and embedded monitoring mechanisms have been proposed.

The four core technical frameworks reviewed herein provide a structured blueprint for the next generation of biosensor systems. In future design and algorithm selection, IPSS principles advocate for co-designing sensor interfaces and preprocessing algorithms to tackle noise and drift at the source. MFE guides the integration of heterogeneous biosignals through architectures capable of learning cross-modal representations, enhancing diagnostic specificity. PDAD methodologies underscore the importance of embedding self-validation and anomaly detection modules within the sensor system itself to ensure data integrity and operational safety over time. Finally, DCEC dictates the adoption of adaptive compression and on-device intelligence to meet the stringent constraints of power, bandwidth, and latency in wearable or implantable scenarios. Together, these frameworks move beyond isolated performance metrics, promoting a holistic, system-level approach to creating robust, intelligent, and practical biosensing solutions.

Looking ahead, the integration of ML and flexible electronics will aim to build an integrated intelligent system with “perception–decision-making–execution” full-chain capabilities. Key directions include developing high-performance flexible devices based on inverse design, biodegradable epidermal electronic systems, and achieving closed-loop diagnosis and treatment; flexible sensing and reinforcement learning energy management strategies for extreme environments; and establishing standardized datasets and testing procedures to promote compliance and industrialization. Through continuous breakthroughs in material, process, and algorithm bottlenecks, strengthening interdisciplinary collaboration, intelligent flexible systems are expected to achieve large-scale applications in personalized medicine, human–computer interaction, and the Internet of Things, reshaping the interaction boundary between electronics, the human body, and the environment, and ushering in a new era of flexible intelligence.

## Figures and Tables

**Table 1:** Summary of key machine learning models and their evaluation metrics in flexible electronics.

Technology Category	Model/Algorithm	Application Task	Key Evaluation Metrics	Ref.
	LSTM	Robot tactile signal classification	Accuracy 98.7% → 99.0% (after 50 training sessions)	[15]
	Improved LSTM + COA	Vehicle network attack identification	Accuracy 98.9%	[16]
IPSS	Attention-LSTM	UWB/INS fusion positioning	Positioning accuracy 0.08–0.17 m	[17]
	CNN	Laryngeal vibration semantic recognition	Accuracy > 99%	[14]
	GRU-LSTM hybrid network	Early hypoglycemia detection in preterm infants	Accuracy 99.6%	[12]
	LSTM	Joint posture recognition	Accuracy 97.13%	[25]
	Neuromorphic computing	Sepsis classification	Accuracy 84.4%	[26]
MFE	PSO-SVM	Vertebral bone layer recognition	Accuracy 90.64%	[28]
	EMO-GCN	Major depressive disorder detection	Accuracy 96.76%	[31]
	CNN	Stress state classification	Accuracy 90.96%	[29]
	SO-YOLO	Chip surface defect detection	mAP 86%	[38]
PDAD	Transformer + CNN	PCB manufacturing defect classification	mAP 98.1%, parameters 7.02 M	[39]
	ATT-YOLO	Surface defect detection of electronic components	mAP 90.3%, inference speed 111 FPS	[41]
	LSTM autoencoder	Anomaly detection in remote patient monitoring	Accuracy 93%, F1-score 0.96	[40]
	CNN-GNN hybrid model	Margin assessment in basal cell carcinoma	Single-case processing time 78 s	[49]
DCEC	Random Forest	High-throughput screening of MOF catalysts	Test accuracy 98.65%	[50]
	Hybrid deep learning compression architecture	Medical image compression	PSNR 50.36 dB, encoding/decoding time 0.065 s	[51]
	TD3 algorithm	Soft exoskeleton walking assistance	Metabolic cost reduction 12.9% ± 3.3%	[52]

**Table 2:** Advancements, pros and cons, and applications of recently developed ML-assisted flexible electronics.

Technology	Advancement and Advantages	Key Challenges	Applications	Refs.
IPSS	improved SNR, dynamic feature extraction, hardware-level compression, strong motion artifact resistance	signal drift, poor multimodal synchronization, environmental noise interference, high hardware integration complexity	robotic tactile sensing, speech recognition, health monitoring	[11,12,13,14,15,17]
MFE	multi-source information fusion, cross-modal feature complementarity, improved classification accuracy, adaptability to complex scenarios	high data heterogeneity, difficult feature alignment, high model complexity, low real-time fusion efficiency	emotion recognition, disease diagnosis, gesture recognition, motion state analysis	[24,25,26,31,32,33]
PDAD	high-resolution imaging, strong capability for small defect detection, real-time monitoring, adaptability to complex industrial environments	difficulty in extracting features of minor defects, strong environmental interference, poor model generalization, high computational resource demand	chip surface defect detection, PCB manufacturing quality inspection, medical anomaly warning	[38,39,40,41,43,44,45]
DCEC	reduced data transmission volume, real-time local processing, low power consumption, data privacy protection	balance between compression loss and accuracy, limited edge device computing power, difficulty in model lightweighting, low multi-device collaboration efficiency	wearable health monitoring, mobile medical diagnosis, smart textiles, industrial IoT sensing	[46,47,48,49,50,51,52,53,54]

**Table 3:** Overview of machine learning-assisted flexible electronics: application domains, integration levels, and key considerations.

Application Domain	Core ML Contribution	Primary Challenge	Cost–Benefit Analysis	Refs.
Wearable Health Monitoring System	Enables precise analysis of physiological signals for disease prediction and rehabilitation assessment.	Signal interference from motion artifacts; Lack of large-scale clinical validation.	Sensor Cost: Moderate to High; Data Processing Cost: Moderate; Benefit: High potential for reducing hospital visits and enabling early intervention.	[12,55,58,61,64,68]
Intelligent Control of Soft Robots	Provides adaptive control and environmental perception for robots in unstructured environments.	Modeling nonlinear dynamics; Real-time sensor fusion; Material durability.	System Cost: High; Development Cost: High; Benefit: Enables robots to operate in complex, human-centric environments.	[76,80,82,85,92]
Performance Optimization of Self-Powered Devices	Optimizes energy harvesting and management, and enhances signal recognition from body-powered sensors.	Stability of energy output (e.g., signal drift); Balance between efficiency and biocompatibility.	Device Cost: Moderate; Lifetime Cost: Low; Benefit: Enables long-term, maintenance-free deployment for IoT and wearable sensing.	[97,101,102,103,105]
Intelligent Perception of Epidermal Electronic Systems	Transforms high-noise, multi-modal skin signals into commands for silent speech interfaces and non-invasive diagnostics.	Long-term signal stability on skin; Generalization of models across users; Clinical translation.	Fabrication Cost: High; Algorithm Development Cost: High; Benefit: Enables revolutionary human–computer interfaces and point-of-care diagnostics.	[109,115,117,121,123]

## Data Availability

No new data were created or analyzed in this study.

## Abbreviations

The following abbreviations are used in this manuscript:

ML	Machine Learning
LSTM	Long Short-Term Memory Network
CNN	Convolutional Neural Network
RL	Reinforcement Learning
IPSS	Intelligent Processing of Sensor Signals
MFE	Multimodal Feature Extraction
PDAD	Process Defect and Anomaly Detection
DCEC	Data Compression and Edge Computing
ResNet	Residual Network
OEST	Organic Electrochemical Synaptic Transistors
COA	Crocodile Optimization Algorithm
GRU	Gated Recurrent Unit
CRO	Chemical Reaction Optimization
LM	Liquid Metal
SVM	Support Vector Machines
NLP	Natural Language Processing
EDA	Electrodermal Activity
ACC	Accelerometer
AOI	Automatic Optical Inspection
ATT-YOLO	Attention Mechanism You Only Look Once
SO-YOLO	Solder Object You Only Look Once
PCBs	Printed Circuit Boards
MLR	Multiple Linear Regression
SBF	Selective Box Fusion
SDWD	Soft Decision Wavelet Decomposition
SVR	Support Vector Regression
EMG	Electromyography
FISA	Flexible Integrated Sensing Array
RF	Random Forest
L-TENG	Length TENG
PPO	Proximal Policy Optimization
IMUs	Inertial Measurement Units
T-DNN	Tandem Deep Neural Network
EBFCs	Enzyme Biofuel Cells
CAM	Relevant Class Activation Map
SNR	Signal-To-Noise Ratio
